# Identification of virus epitopes and reactive T-cell receptors from memory T cells without peptide synthesis

**DOI:** 10.1038/s42003-024-07048-x

**Published:** 2024-11-04

**Authors:** Lihui Wang, Runda Xu, Daosheng Huang, Pai Peng, Keyong Sun, Jie Hu, Bei-zhong Liu, Liang Fang, Liwen Zhang, Xin Sun, Fei Gu, Ni Tang, Ai-long Huang, Xin Lin, Xun Lan

**Affiliations:** 1 Department of Basic Medical Science, School of Medicine, Tsinghua University, 100084 Beijing, China; 2 Tsinghua-Peking Joint Center for Life Sciences, Tsinghua University, 100084 Beijing, China; 3 MOE Key Laboratory of Bioinformatics, Tsinghua University, 100084 Beijing, China; 4 Key Laboratory of Molecular Biology for Infectious Diseases (Ministry of Education), Institute for Viral Hepatitis, Department of Infectious Diseases, the Second Affiliated Hospital, Chongqing Medical University, 400016 Chongqing, China; 5 Yong-Chuan Hospital of Chongqing Medical University, Chongqing, China; 6 Alibaba Group, 311121 Hangzhou, China

**Keywords:** Cellular immunity, Immunological techniques, SARS-CoV-2

## Abstract

Identifying epitopes and their corresponding T-cell receptor (TCR) sequences is crucial in the face of rapidly mutating viruses. Peptide synthesis is often required to confirm the exact epitope sequences, which is time-consuming and expensive. In this study, we introduce a scalable workflow to identify the exact sequences of virus epitopes and reactive TCRs targeting the epitopes from memory T cells. Following the narrowing down of epitopes to specific regions via the tandem minigene (TMG) system, our workflow incorporates the utilization of peptide-major histocompatibility complex-displaying yeasts (pMHC-displaying yeasts) to rapidly screen immunogenic epitopes’ precise sequences, obviating the necessity for the chemical synthesis of peptides. Focusing on SARS-CoV-2, we identify the precise sequences of reactive TCRs, targeting conserved epitopes across the Coronaviridae family, from the blood of COVID-19-recovered individuals over 8 months. Notably, we reveal that at least 75% (6/8) of the tested donors harbor T cells targeting a shared epitope, KTFPPTEPK, derived from the N protein. Furthermore, several identified TCRs exhibit cross-reactivity to mutant epitopes, suggesting a potential mechanism for sustained T-cell responses against emerging SARS-CoV-2 variants.

## Abstract (web summary)

A scalable workflow involving a pMHC-displaying yeast system is introduced to identify precise virus epitope sequences and corresponding T-cell receptor (TCR) sequences from memory T cells.

## Introduction

The emergence of several variants of SARS-CoV-2, including Alpha (B.1.1.7), Beta (B.1.351)^1^, Gamma (P.1), Delta (B.1.617.2)^2^, and the recent Omicron (B.1.1.529)^3^, has raised significant concerns about immune evasion among vaccinated or previously infected populations. Notably, the Omicron variant has drawn attention due to its potential to evade existing neutralizing antibodies^4–10^, primarily attributable to its high mutational load within the receptor-binding domain (RBD) of the Spike (S) protein, which is a key target of antibody responses. However, studies have indicated that T-cell responses exhibit cross-reactivity against numerous SARS-CoV-2 variants, including Omicron, suggesting a potential avenue for sustained immune protection^11–14^. Longitudinal investigations have underscored the durability of T-cell immunity against SARS-CoV-2, persisting for over a year post-infection^15–20^, contrasting the reduced antibody responses observed over time^15,21,22^. This highlights the critical role of SARS-CoV-2-specific T-cell immunity in long-term immune surveillance.

Efforts to identify the epitopes inducing sustained T-cell responses to SARS-CoV-2 have led to the identification of specific peptide targets^23–26^, some of which have been employed in vaccine development to elicit T-cell-mediated immunity^27^. Currently, existing methods for identifying T-cell epitopes predominantly rely on synthetic peptide libraries for screening. These approaches typically require the synthesis of peptide libraries either through the prediction of peptide sequences or the enumeration of overlapping peptides. After in vitro stimulation via peptide pulsing, a variety of techniques, including enzyme-linked immune absorbent spot (ELISpot), intracellular cytokine staining (ICS), activation-induced markers (AIMs), and multimers, are employed to identify T-cell epitopes^17,26,28–32^. While this methodology is well-established, it presents inherent limitations. Notably, the necessity to test peptides of varying lengths, typically ranging from 8 to 11 amino acids, contributes to the high cost and time-consuming nature of peptide synthesis. Furthermore, the processing of peptides, including proteolytic cleavage within cells, is often overlooked. As a result, a peptide that can be loaded onto MHCs via peptide pulsing may not be produced and presented effectively due to the bias of intracellular proteolytic cleavage.

In tumor neoantigen identification, minigene construction is frequently employed to pinpoint immunogenic neoantigens^33,34^. This technique involves cells expressing multiple predicted epitopes encoded by tandem minigenes, effectively simulating in vivo peptide processing and presentation. Nonetheless, this method encounters a significant challenge: the precise sequences of the presented peptides remain elusive, primarily due to the uncertainty of proteolytic cleavage sites.

In this study, we present a scalable workflow aimed at identifying TCRs targeting virus epitopes derived from memory T cells, with no need for peptide synthesis. Our approach integrates the TMG system with a pMHC-displaying yeast system, facilitating rapid confirmation of precise peptide screening and evaluation of cross-reactivity to mutant peptides. Employing a proof-of-principle study focusing on SARS-CoV-2, we identified epitopes conserved across the Coronaviridae family. Furthermore, we delineated the exact TCR sequences of reactive T cells from the blood of COVID-19-recovered individuals, spanning up to 8 months post-infection. Remarkably, these identified TCRs demonstrated cross-reactivity to mutant antigens, suggesting their potential role in sustaining T-cell responses against evolving SARS-CoV-2 variants.

## Results

### A scalable workflow for identifying epitope–TCR pairs from memory T cells

**Fig. 1:**
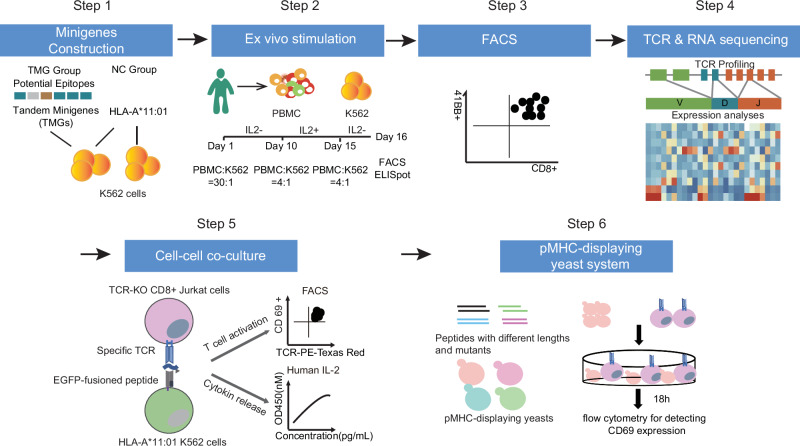
The workflow for rapid epitope and corresponding T-cell receptor (TCR) sequence identification. Peptides from the IEDB database or predicted by NetMHCpan 4.1 were regarded as potential epitopes, followed by the construction of tandem minigenes, each encoding 9 or 10 potential epitopes flanked by 5-amino-acid sequences (Step 1). To isolate TCRs from long-term memory T cells, epitope-specific T cells were expanded ex vivo by co-culturing PBMCs with TMG-expressing K562 cells and a single HLA allele for 16 days (Step 2). Activated T cells (4-1BB+) were then sorted (Step 3) and subjected to single-cell TCR sequencing (Step 4). To establish epitope-TCR pairs, single epitopes fused with an enhanced green fluorescent protein (EGFP) were expressed in K562 cells alongside a single HLA allele. Reactivity was assessed using TCR-KO CD8+ Jurkat T cells through FACS and enzyme-linked immunoassay (ELISA) (Step 5). Precise epitope lengths and mutant peptides were determined by engineering yeasts to express peptides covalently linked to HLA molecules. T-cell activation was assessed by measuring CD69 expression via FACS (Step 6). This workflow enables the identification of epitope sequences and their reactive TCRs from long-term memory T cells without the need for peptide chemical synthesis.

To expedite the identification of epitopes and their corresponding T-cell receptor (TCR) sequences, we established a scalable workflow. First, recorded peptides presented by human leukocyte antigens (HLAs) from the IEDB database^35^ or predicted peptides from NetMHCpan 4.1 were regarded as potential epitopes. Next, 9 or 10 potential epitopes were encoded by a minigene in tandem, with each epitope flanked by a 5-amino-acid sequence on each side. To isolate TCRs from memory T cells, epitope-specific T cells were ex vivo expanded by co-culturing peripheral blood mononuclear cells (PBMCs) with K562 cells expressing TMGs and HLA-A*11:01 allele for 16 days. The expanded cells were then collected and sorted for 4-1BB+ T cells, indicative of activated T cells. Subsequent single-cell TCR sequencing facilitated the identification of TCR sequences (Fig. 1).

Establishing epitope–TCR pairs involved expressing a single epitope fused with an enhanced green fluorescent protein (EGFP) in K562 cells expressing a single HLA allele. The reactive TCRs were introduced into TCR-knockout (TCR-KO) CD8+ Jurkat T cells. Co-culturing T cells and K562 cells followed by analysis via fluorescence-activated cell sorting (FACS) and enzyme-linked immunoassay (ELISA) allowed for the evaluation of the T-cell response.

To determine the precise length and sequences of immunogenic epitopes, we engineered yeasts to express peptides of varying lengths covalently linked to HLA molecules. Co-culturing these engineered yeasts with TCR-KO CD8+ Jurkat T cells enabled the activation of T cells solely by the correct peptide, as measured by CD69 expression detected via FACS. Utilizing Golden Gate Assembly, different yeast libraries expressing mutant peptides could be rapidly constructed.

In conclusion, this streamlined workflow enables the identification of exact epitope sequences and their corresponding reactive TCRs from memory T cells, bypassing the need for chemical synthesis of peptides.

### Potential shared epitopes among coronaviruses for minigene construction

As an RNA virus, SARS-CoV-2 exhibits a high mutation rate and may facilitate evasion of vaccines^36^. Identifying TCR sequences targeting conserved epitopes and their variants presents a valuable resource for studying T cell immunity. Here, we conducted a proof-of-principle study focusing on SARS-CoV-2.

**Fig. 2:**
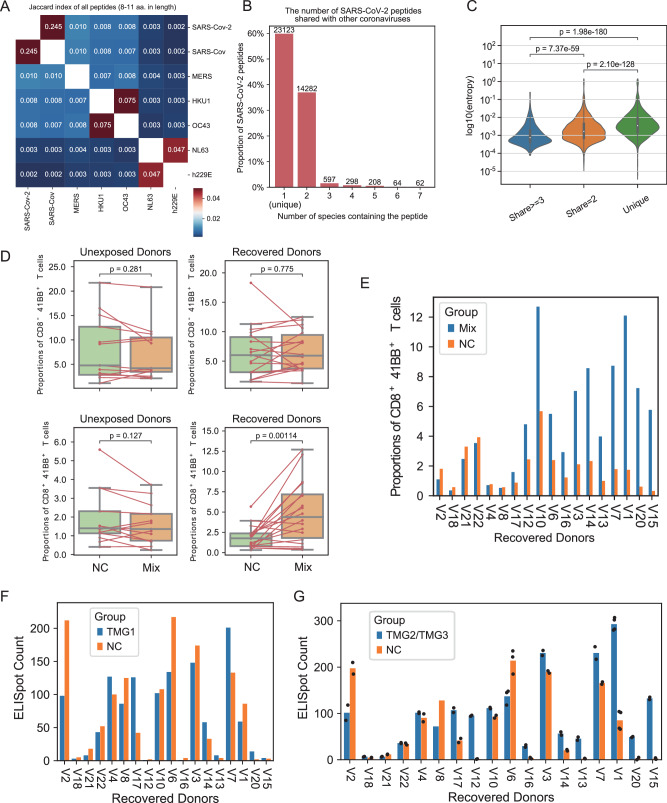
Stimulation experiments of T cells from recovered donors and unexposed donors. **A** Heatmap showing the Jaccard index of all peptides with 8–11 amino acids (aa.). **B** The number of SARS-CoV-2 peptides shared with other coronaviruses. **C** The entropy of each amino acid site in different groups. (Wilcoxon rank-sum test). **D** Boxplots showing the proportions of cells in different quadrants. Mix: PBMC co-cultured with K562 cells carrying TMG1, TMG2, and TMG3; NC negative control, PBMC co-cultured with K562 without TMG (paired *t*-test, *n* = 13 biologically independent donors for unexposed donors; *n* = 18 biologically independent donors for recovered donors). **E** Barplot showing the proportions of CD8^+^ 41BB^+^ T cells for samples from recovered donors. One experiment was conducted for each condition. **F** The results of ELISpot for co-culture with TMG1. One experiment was conducted for each condition. **G** The results of ELISpot for co-culture with mixed TMG2/TMG3. The number of technical replicates was decided for each condition according to the amount of available PBMCs. Each dot represents one replicate. V8 has no replicate due to insufficient PBMCs; For V6, *n* = 3 technical replicates; For V1, *n* = 4 technical replicates; For other donors, *n* = 2 technical replicates.

We compiled proteomes from seven different coronaviruses: SARS-CoV-2 (WIV04), SARS-CoV, Middle East respiratory syndrome (MERS), human coronavirus-229E, -OC43, -NL63, and -HKU1 to identify conserved peptide sequences. We enumerated all possible peptides ranging from 8 to 11 amino acids from each virus, generating seven peptidomes. The Jaccard index between the peptidomes revealed high similarity between SARS-CoV-2 and SARS-CoV (Fig. 2A). Subsequently, we categorized the 38,634 peptides from SARS-CoV-2 into three conservation groups: (1) 23,123 peptides unique to the virus (Unique); (2) 14,282 peptides shared with another virus species (Share = 2); and (3) 1229 peptides shared among three or more virus species (Share ≥ 3) (Fig. 2B).

To assess the conservation degree of peptides within these three groups, we gathered 6,683,868 recorded sequences of SARS-CoV-2 variants from Global Initiative on Sharing All Influenza Data (GISAID^37^) and analyzed the cumulative distribution of minor allele frequency for amino acid sites in these peptides (Fig. S1A). Additionally, we computed the entropy for each amino acid site, with those in the “Share ≥ 3” group exhibiting low average entropy, indicative of high conservation (Fig. 2C). Site-specific entropy for different virus proteins is presented in Fig. S1B, C. Our analysis revealed that amino acid sites in the “Share ≥ 3” group were the most conserved, while those in the “Unique” group were the least conserved.

Utilizing NetMHCpan 4.1 and the IEDB database, we collected potential presented peptides from SARS-CoV-2 for the HLA allele A*11:01, the most prevalent HLA-A allele among Chinese individuals^38^. According to the conservation level among different coronaviruses, we selected 28 peptides to be loaded into three tandem minigenes (TMGs) alongside flanking sequences (Supplementary Data 2). TMG1 and TMG2 encompassed peptides from nonstructural proteins (NSPs), whereas TMG3 contained peptides from the Spike, M, and N proteins. These three TMGs were separately transfected into K562 cells exclusively expressing HLA-A*11:01.

### Expansion of SARS-CoV-2-specific T cells from donors 7–8 months post-COVID-19 infection

To assess long-term T-cell immunity against SARS-CoV-2, we specifically targeted donors carrying the HLA-A*11:01 allele. PBMCs were collected from 13 individuals unexposed to COVID-19 and 18 recovered donors at 7–8 months post-infection (Supplementary Data 3).

Epitope-specific T cells were ex vivo expanded by co-culturing PBMCs with TMG-expressing K562 cells over 16 days. In the experimental setup, a combination of K562 cells carrying TMG1, TMG2, and TMG3 (hereafter referred to as TMGmix) was employed. Conversely, in the negative control (NC) group, K562 cells expressing HLA-A*11:01 (K562-1101) but lacking TMG were used.

FACS analysis revealed significantly higher proportions of CD8+ 4-1BB+ T cells in TMGmix groups compared to NC groups (Figs. 2D, S2, and S3). Notably, expansions of CD8+ 4-1BB+ T cells were observed in the majority of recovered donors (12 out of 18, 66.7%), indicating the persistence of SARS-CoV-2-specific CD8+ T cells for up to 8 months (Fig. 2E, S4B–D).

Further elucidation of the TMG responsible for expansion in TMGmix groups was evaluated through ELISpot assays to measure the release of IFN-γ by T cells (Fig. S4A). Almost no activation was detected when co-culturing PBMCs with TMG1 (Fig. 2F). Considering the insufficient donors’ PBMCs, we next co-cultured them with a mix of TMG2 and TMG3 with 1–4 replicates according to the amount of available PBMCs (Fig. 2G). The results were consistent with the upregulation of surface 41BB in Fig. 2E and indicated that antigens within TMG2 or TMG3 were mainly responsible for T-cell activation.

### TCR profiling and expression analysis of SARS-CoV-2-specific T cells

**Fig. 3:**
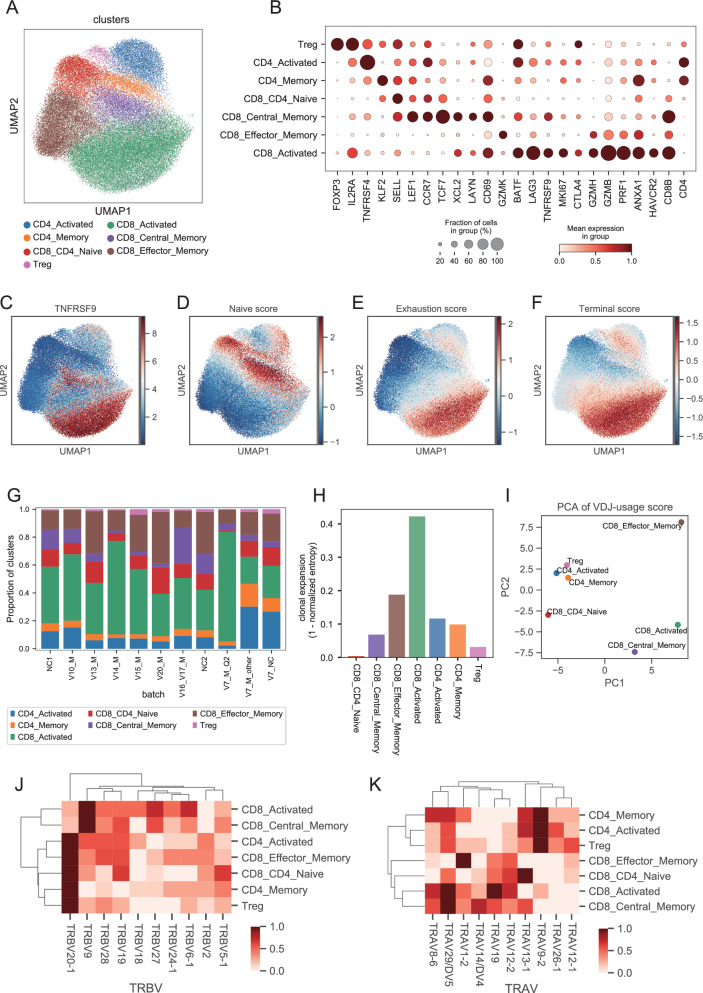
Analysis of single-cell transcriptome and TCR information. **A** UMAP showing the T-cell clusters based on scRNA-seq. **B** Dot plot showing the marker genes of T-cell clusters. TNFRSF9 is the gene symbol of 41BB (CD137). **C** The expression of TNFRSF9 on the UMAP. Values indicate normalized counts. **D**–**F** UMAPs showing the Naive score (**D**), Exhaustion score (**E**), and Terminal score (**F**). **G** The distributions of clusters in each sample batch. **H** Cells in CD8_Activated cluster showed the highest clonal expansion calculated by 1-normalized entropy. **I** The first two components of PCA based on VDJ usage scores. **J** and **K** Heatmap showing the VDJ-usage scores of top-10 TRBV genes (**J**) and of top-10 TRAV genes (**K**).

T cells with significant activation in the ELISpot experiments were collected for single-cell sequencing. T cells were sorted via FACS, and cells from different quadrants were mixed at specific ratios to control the distribution of cell types (Table S1). Eventually, T cells from 8 donors (V7, V10, V13, V14, V15, V16, V17, and V20) were successfully profiled by single-cell TCR sequencing (scTCR-seq) and single-cell RNA sequencing (scRNA-seq). Following quality control measures, a total of 68,124 T cells with both transcriptome and TCR information were obtained. These cells were clustered into 7 distinct groups (Fig. 3A): naive CD8+ or CD4+ T cells (CD8_CD4_Naive), CD4+ memory T cells (CD4_Memory), CD4+-activated T cells (CD4_Activated), Regulatory T cells (Tregs), CD8+ effector memory T cells (CD8_Effector_Memory), CD8+ central memory T cells (CD8_Central_Memory), and CD8+-activated T cells (CD8_Activated).

Among these clusters, CD8_Activated and CD8_Central_Memory exhibited high expression of *TNFRSF9* (4-1BB) (Fig. 3B, C), which served as the activation marker in FACS. Furthermore, CD8_Central_Memory displayed elevated naive scores as measured by *TCF7*, *LEF1*, and *SELL* (Fig. 3B, D), while CD8_Activated exhibited heightened exhausted scores measured by *CTLA4*, *HAVCR2*, *LAYN*, *LAG3*, and *TIGIT* (Fig. 3B, E). A terminally differentiated score (terminal score) was obtained by subtracting the naive score from the exhausted score (Fig. 3F). The pronounced exhaustion of activated CD8+ cells likely stemmed from continuous stimulation by viral antigens. The distribution of cell clusters was balanced across each sample and aligned with the mixing ratios of T cells from different quadrants (Fig. 3G and Table S1).

T cells within CD8_Activated exhibited the most significant clonal expansion, as measured by one minus normalized entropy (Fig. 3H, see the “Methods” section). VDJ gene usage of TCRs displayed biases across different cell subsets. CD4_Activated, CD4_Memory, and Tregs displayed similar VDJ biases, while the VDJ bias of CD8_Activated closely resembled that of CD8_Central_Memory (Fig. 3I–K), consistent with the expression pattern of *TNFRSF9* (Fig. 3C). This suggests that SARS-CoV-2-specific activated T cells are more likely derived from central memory T cells.

### Identification and analyses of responsive TCRs

**Fig. 4:**
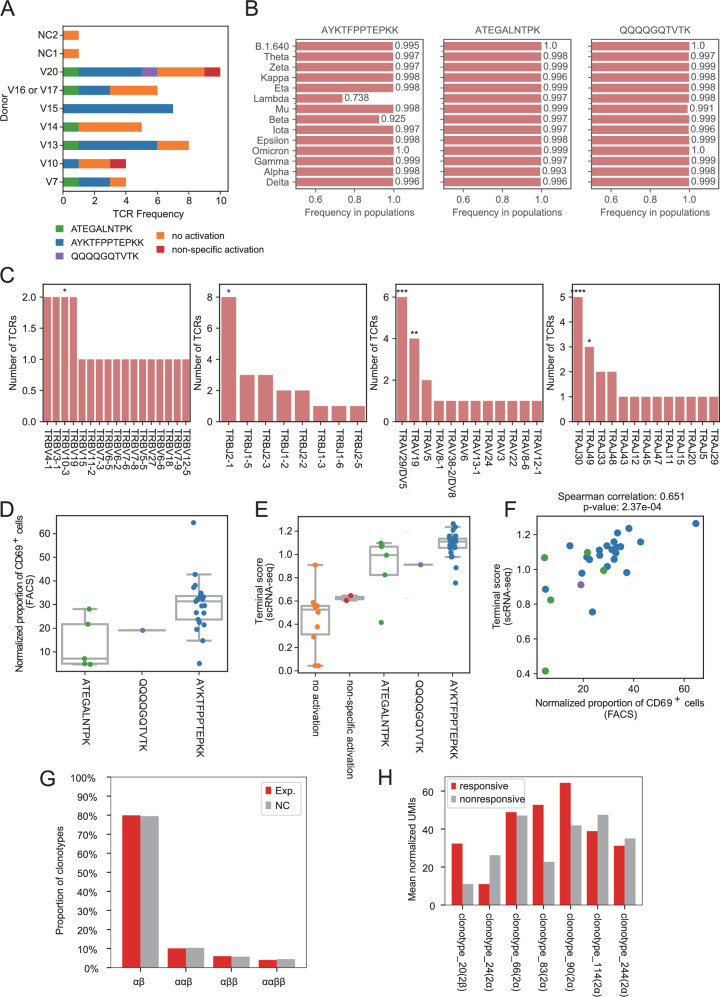
Identifying specific TCR sequences targeting SARS-CoV-2 epitopes. **A** The distribution of identified targets of tested TCRs in each donor group. “V16 or V17” indicates the group contains cells from both V16 and V17. “NC1” and ”NC2” indicate negative control groups. See Table S1 for more details. **B** Wild-type frequency of three identified epitopes in different SARS-CoV-2 variants. **C** The distribution of TRBV, TRBJ, TRAV, and TRAJ gene usage of 21 TCRs targeting AYKTFPPTEPKK. TRBV10-3: *p* = 4.63e−2, TRBJ2-1: *p* = 2.25e−2, TRAV29/DV5: *p* = 5.86e−4, TRAV19: *p* = 8.50e−3, TRAJ30: *p* = 8.42e−5, TRAJ49: *p* = 4.12e−2 (Fisher’s exact test). **D** Normalized proportions of CD69^+^ cells of Jurkat T cells with different TCRs in FACS. Each dot indicates the mean value of results from three independent experiments. The normalized proportions were calculated by subtracting the proportions in negative control groups from those in experimental groups. **E** Terminal scores of cells with different TCRs in scRNA-seq data. Each dot represents the mean value of T cells with a specific TCR. **F** Scatter plot showing the correlation between normalized proportions of CD69^+^ T cells and terminal scores of cells. Each dot represents the mean value of T cells with a specific TCR. The colors of the dots indicate the epitope in (**D**). **G** Distributions of clonotypes with different numbers of α and β chains in the experimental group (Exp.) and the negative control group (NC). **H** Mean normalized UMIs of α or β chains in tested clonotypes with allelic inclusion.

We selected 39 clonal expanded T-cell clonotypes with complete α and β chain sequences from CD8_Activated cells, including 2 clonotypes from control groups (Fig. 4A and Supplementary Data 4). Seven of these clonotypes contained 2 α chains or 2 β chains; thus, a total of 46 TCR sequences were separately transduced into Jurkat T cells. Each antigen from TMG2 and TMG3 was reconstructed into individual vectors fused with EGFP and transfected into K562 cells expressing HLA-A*11:01 (K562-1101). A total of 874 combinations of TCRs and antigens were co-cultured in pairs. Subsequently, T cells were analyzed by FACS and ELISA. Using CD69 as the marker of activated Jurkat T cells, we identified 27 cognate TCRs targeting epitopes of SARS-CoV-2 (Fig. 4A, Supplementary Data 4): 21 of the TCRs from 6 donors were activated by AYKTFPPTEPKK; 5 of the TCRs from 5 donors were activated by ATEGALNTPK; 1 of the TCRs was activated by QQQQGQTVTK. All three epitopes came from the N protein of SARS-CoV-2 and were shared by SARS-CoV. Peptide conservation analysis across different variants of SARS-CoV-2 revealed that 26% of Lambda variants had a T366I mutation, while 7% of Beta variants had a T362I mutation in AYKTFPPTEPKK (Fig. 4B).

We investigated the VDJ gene usage of 21 TCRs targeting AYKTFPPTEPKK (Fig. 4C). Fisher’s exact test was performed for each VDJ gene in this group with the VDJ usage in all clonotypes as the background. The frequencies of TRAV29/DV5 and TRAJ30 were significantly higher than that of the background, which was consistent with their enrichment in CD8_Activated and CD8_Central_Memory (Fig. 3K, Fig. S5A–D).

Analysis of the relationship between T-cell responsiveness and transcriptome profile revealed that TCRs with larger normalized proportions of CD69+ T cells were associated with higher terminal scores in scRNA-seq data (Fig. 4D–F). The activation levels were further measured by the release of interleukin-2 (IL-2) in ELISA, showing a positive correlation with terminal scores in scRNA-seq data at the TCR level (Fig. S5E-F). Considering that the measurements of IL-2 and CD69^+^ T cells were based on TCR-engineered Jurkat cell lines, while the scRNA-seq data were derived from the donor’s blood, it indicated that the TCR sequence was an important factor in determining the responsiveness of T cells to a specific epitope.

There were approximately 20% of clonotypes had multiple TCRs (Fig. 4G), which accounted for 15% of T cells (Fig. S5G). Besides, 18% (7/39) of our experimentally tested clonotypes had dual TCR, and 100% (7/7) of them turned out to have one SARS-CoV-2-responsive TCR (Supplementary Data 4). As 100% (7/7) of these clonotypes had only one responsive TCR, we wondered whether the expression of α or β chains could indicate functionality. Although the α or β chain with higher expression was often regarded as the functional chain, our results showed that the responsive TCRs were not always made of chains with higher expression (Fig. 4H).

### The cross-reactivity of TCRs and identifying the exact epitopes using the pMHC-displaying yeast system

**Fig. 5:**
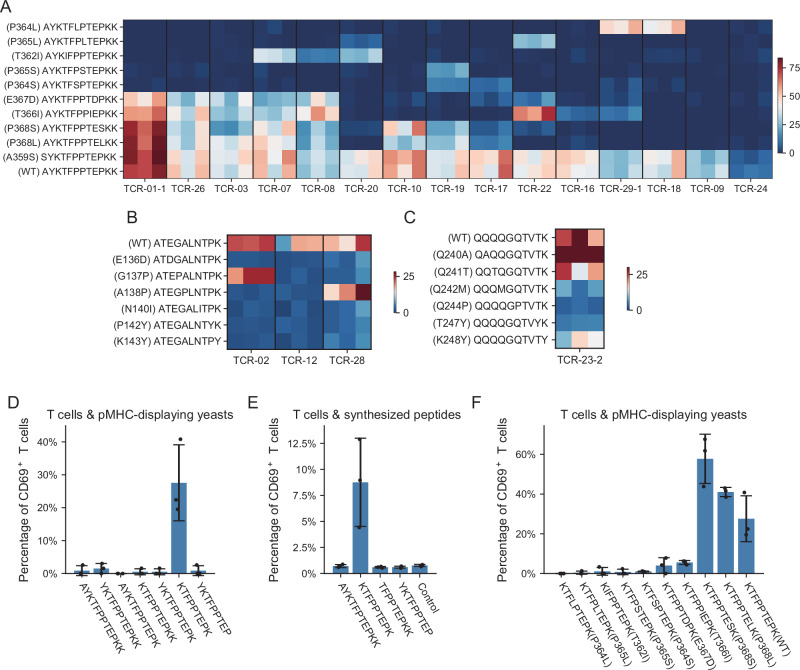
Cross-reactivity of TCRs against mutant peptides. **A**–**C** Heatmap showing the normalized proportions of CD69^+^ T cells after co-cultured with K562 cells carrying different mutant peptides, indicating the cross-reactivity of TCRs against mutant AYKTFPPTEPKK (**A**), ATEGALNTPK (**B**), and QQQQGQTVTK (**C**). Three replicates for each TCR-peptide pair are shown. **D** The exact epitope sequence of the presented peptide within AYKTFPPTEPKK was detected by the pMHC-displaying yeast system, co-cultured with TCR-01-1 Jurkat cells. **E** The exact epitope sequence of the presented peptide within AYKTFPPTEPKK was detected through peptide-loaded K562-1101 cells, co-cultured with TCR-01-1 Jurkat cells. Control indicates no peptide was loaded to K562-1101 cells. **F** The cross-reactivity of TCRs to mutant epitopes detected by the pMHC-displaying yeast system. For **D–F**, *n* = 3 biological replicates for each condition, and the mean ± standard deviation (SD) is shown as error bars.

To investigate the cross-reactivity of these TCRs to mutant epitopes, we introduced point mutations to the peptides and measured T-cell responses through co-culture experiments. The normalized proportions of CD69^+^ T cells from three replicates are shown in Fig. 5A–C. Some of the TCRs were able to recognize multiple mutant peptides, including known mutants of T366I and T362I in the Lambda and Beta variants, respectively. Among these TCRs, TCR-01-1 exhibited dramatic responses to the wild-type peptide as well as many of the mutant peptides. Moreover, T cells expressing TCR-01-1 gained the highest terminally differentiated score in scRNA-seq data. These findings suggest that TCR-01-01 may possess greater sensitivity to the viral antigen than other TCRs. Additionally, the response of TCR-23-2 to the Q240A mutant appeared higher than that of the wild type.

To identify the exact sequences of epitopes that were recognized by T cells, we engineered a pMHC-displaying yeast system. These yeast cells expressed peptides of various lengths derived from AYKTFPPTEPKK, covalently linked to HLA-A*11:01 molecules. We co-cultured these yeasts with TCR-01-expressing CD8+ Jurkat T cells and measured the CD69 expression by FACS as an indicator of T-cell activation. Remarkably, only KTFPPTEPK was able to activate the T cell (Figs. 5D and S6), demonstrating KTFPPTEPK as the exact epitope. To further verify that KTFPPTEPK did activate the T cells, we used synthesized peptides AYKTFPPTEPKK, KTFPPTEPK, YKTFPPTEP, and TFPPTEPKK and loaded them onto K562-1101 cells to activate T cells expressing the TCR-01-1 TCR. The results verified that only KTFPPTEPK could activate the T cells (Figs. 5E and S6C).

We also assessed the cross-reactivity with the exact peptides displayed on yeasts (Fig. 5F), and the results were consistent with that of K562 cells expressing minigenes (Fig. 5A). This underscores the potential of the pMHC-displaying yeast system as a cost-effective and rapid approach compared to chemical peptide synthesis for identifying exact epitope sequences and evaluating cross-reactivity.

## Discussion

In this study, we provided a scalable workflow to identify SARS-CoV-2-specific TCRs and their epitopes from recovered individuals 7–8 months after the infection of COVID-19. While prior research has documented the long-term persistence of memory T cells^15–20^, most studies utilized a synthesized SARS-CoV-2 peptide pool to stimulate T cells. Unlike the conventional approach of directly adding peptides to culture media, our method circumvents the need for chemical peptide synthesis by combining the TMG system and pMHC-displaying yeast system. The TMG system not only narrowed down the range of potential epitopes but also ensured that the peptides were endogenously produced by cells and presented on HLA molecules via the natural antigen processing pathway, involving proteasomal cleavage and transportation into the endoplasmic reticulum. To identify the exact epitope sequences, which cannot be provided by the TMG system, we utilized a pMHC-displaying yeast system to obtain the precise epitopes in a cost-effective way, which showed similar results to peptide pulsing.

Remarkably, 21/27 of the responsive TCRs from 6/8 of the donors can recognize epitopes derived from AYKTFPPTEPKK, and most of them exhibited cross-reactivity to mutant peptides. A previous study has proved that the peptide-based vaccine, CoVac-1, can induce SARS-CoV-2-specific T-cell responses in the phase I open-label trial^27^. Given the widespread recognition of AYKTFPPTEPKK by various donors, it emerges as a promising candidate for T-cell peptide vaccines. Our workflow can be further leveraged to explore dominant epitopes presented by other HLA types, thereby furnishing additional candidates for T-cell peptide vaccines expeditiously. Meanwhile, we identified several identified TCRs that exhibit cross-reactivity to mutant antigens, which can be used in the TCR-T-based therapy of patients with COVID-19 infection. Compared to monoclonal antibodies and antiviral drugs, T-cell immunity has demonstrated good persistence and conservatism, which is of significant importance for adaptive immunotherapy, especially in treating immunocompromised individuals. Currently, SARS-CoV-2 TCR-T has proven beneficial in the treatment of COVID-19 in transplant patients who are on long-term immunosuppressants^39^.

Although our research was only validated with HLA-A*11:01 and a small number of donors, it served as a proof-of-principle study in elucidating the exact sequences of SARS-CoV-2-specific TCRs, their precise epitopes, and cross-reactivity. This offers valuable insights into long-term T-cell responses to SARS-CoV-2 and TCR–pMHC interactions.

## Methods

### Ethics statement

This project was approved by the ethics committee of Tsinghua University (Project No. 20210030) and Chongqing Medical University (Project No. 2021066). Informed consents were obtained from all participants. All ethical regulations relevant to human research participants were followed. This study does not involve race, ethnicity, or other socially relevant groupings. The recovered donors and unexposed donors were recruited randomly in this study. Investigators were blinded to donor identities. The gender data of all donors enrolled in the article was collected; for details, see Supplementary Data 3.

### Data collection

The proteome of SARS-CoV-2 WIV04 and 6,683,868 recorded sequences of SARS-CoV-2 variants were downloaded from GISAID. The proteomes of SARS-CoV, MERS, Human Coronavirus-229E, -OC43, -NL63, and -HKU1 were downloaded from NCBI Virus with accession numbers NC_004718.3, NC_019843.3, NC_002645.1, NC_006213.1, NC_005831.2, NC_006577.2, respectively.

### Entropy calculation

For the entropy of the amino acid site, protein sequences of all variants were aligned to the proteins of SARS-CoV-2 WIV04. For a given site on a specific protein, we used the proportion of each observed amino acid to calculate the Shannon entropy (*H*) as follows:

$$H=-{\sum }_{i=1}^{n}{p}_{i}\times {\mathrm{ln}}\left(p\right)$$

In the equation, *n* is the number of observed amino acids at the site and *p*_*i*_ indicates the proportion of a specific amino acid. Low entropy suggests high conservation.

For evaluating the clonal expansion, the proportions of clonotypes in a given cluster were used to calculate the Shannon entropy. The Shannon entropy was divided by ln(number of clonotypes) to get the normalized entropy. We took (1–normalized entropy) to evaluate the clonal expansion, with a higher value indicating higher dominations by several clonotypes, which suggests stronger clonal expansion.

### Peptide selection and tandem minigene design

Several epitopes from SARS-CoV have been reported to be presented by HLA-A*11:01, which share the same sequence with SARS-CoV-2. Therefore, we collected these peptides recorded in the IEDB database. Next, for more potential epitopes, all possible peptides with 8–11 amino acids were enumerated as the candidate pool, and NetMHCpan 4.1 was used to predict the probability of peptide presented by HLA-A*11:01. The results of the prediction were listed in Supplementary Data 1. We ranked the positive predictions from NetMHCpan by “EL_Rank” and took the top unreported peptides together with the peptides recorded in IEDB for the following tandem minigene design. Reported and predicted peptides shared by more than two types of coronaviruses were used for TMG1. All peptides in TMG1 were from NSPs coincidently. For the other peptides, we took 10 from NSPs and 9 from Spike, M, or N proteins to construct TMG2 and TMG3, respectively. The three TMGs were constructed separately into the lentivirus plasmid pHAGE backbone with a puromycin resistance gene. Each epitope includes a 5-amino-acid flanking sequence on each side, according to a published study^40^. The order of different peptides within the TMG is random.

### K562 cell lines expressing SARS-CoV-2 antigens

To stimulate SARS-CoV-2 antigen-specific T cells from PBMC donors, we first construct K562 cells expressing a single HLA allele-HLA-A*11:01 derived from the K562 cell line (ATCC CCL-243). The construction process is outlined below. The lentiviral pHAGE-IRES-BSD vector containing the HLA-A genes was cloned and produced in Lenti-293X cells. Lentiviruses encoding HLA-A genes were then generated through transient transfection of lentiviral-based vectors and their packaging vectors (psPAX2 and pMD2.G) into Lenti-293X cells. After 48 h of transfection, the virus supernatant was collected and filtered through a 0.45-μm syringe filter. K562 cells were transduced with lentivirus encoding HLA-A*11:01 genes, followed by selection with blasticidin (5 μg/mL) for stable integration of HLA-A*11:01 genes over 14 days. Subsequently, tandem minigenes encoding 20 SARS-CoV-2 epitopes were introduced into K562-HLA- A*11:01 cells via lentiviral infection. The cells were then selected with puromycin (2 μg/mL) for 7 days to enrich for cells expressing the SARS-CoV-2 epitopes.

To validate the functionality of SARS-CoV-2 epitope-specific TCRs, we individually overexpressed 19 single SARS-CoV-2 epitopes fused to the N-terminal of GFP in K562-HLA-A*11:01 cells. After 48 h post-infection, GFP-positive cells were isolated via FACS to ensure the specificity and purity of the antigen-expressing cells.

### T cells construction

For the construction of T cells with 46 different candidate TCRs, we firstly synthesized the DNA sequence of TCRα and TCRβ from TSINGKE, China. These genes were cloned into a lentiviral pHAGE-IRES-RFP vector (a gift from Dr. Xin Lin, Tsinghua University, Beijing, China) as the format TCRβ-Cβ-P2A-TCRα-Cα using the Golden Gate cloning strategy according to the 2X Seamless Clone Kit (Beyotime Biotechnology) protocol.

The lentiviruses encoding these TCRs used for infection of the Jurkat cells were produced in L-293T cells by transient transfection of lentiviral-based vectors and their packaging vectors (psPAX2 and pMD2.G). The viral supernatant was collected at 48 h after transfection, followed by filtering through a 0.45-µm syringe filter.

The Jurkat cells were spin-infected with viral supernatant at 1500 rpm at 32 °C for 90 min. On day 3 post-infection, the transfected Jurkat cells were sorted by Arial SORP flow cytometer and cultured to be used for the following co-culture assay. The L-293T cells and the Jurkat cell with the native TCR deletion were also gifts from Dr. Xin Lin at Tsinghua University, Beijing, China.

### Cell culture

Jurkat clone 5 (JC5) cells were derived from Jurkat E6.1 cells (ATCC TIB-152) by knocking out TCRα and TCRβ chains with a CRISPR/Cas9 system (gRNA sequences: TRBC_GGGCTCAAACACAGCGACCTC, TRAC_GTCTCTCAGCTGGTACACGGC). K562 cell line (ATCC CCL-243) was used to express HLA-A*1101 and the epitopes. These cell lines were constructed by Dr. Xin Lin’s lab at Tsinghua University, Beijing, China. These original cell lines were authenticated by ATCC.L-293T cells were cultured in Dulbecco’s modified Eagle’s medium (DMEM, Gibco, USA) supplemented with 10% fetal bovine serum (FBS, BI), 100 IU/ml penicillin, and 100 μg/ml streptomycin (Gibco, USA). K562 cells and Jurkat Cells were cultured in RPMI-1640 (Gibco, USA) with 10% FBS(BI), 0.1 mM MEM NEAA (Gibco, USA), 1 mM sodium pyruvate (Gibco, USA), and 100 IU 100 IU/ml penicillin and 100 μg/ml streptomycin. All cells were cultured at 37 °C in an incubator with 5% CO_2_. PBMC was cultured in the RPMI-1640 (Gibco, USA) with 10% heat-inactivated FBS (Gibco, USA), 0.1 mM MEM NEAA (Gibco, USA), 1 mM sodium pyruvate (Gibco, USA), and 100 IU 100 IU/ml penicillin and 100 μg/ml streptomycin. Different concentrations of IL-2 (SI HUAN SHENG WU, China) were supplemented into the PBMC culture medium. Mycoplasma contamination was negative based on the PCR results using the supernatant of the culture medium.

### HLA type confirmation

To identify the HLA-A type of the COVID-19 recovered donors and unexposed donors, we extracted the genome using the 100 μl blood samples of the recovered donors and unexposed donors. And the PCR was performed to amplify the HLA-A molecules using the primer F- GAAACSGCCTCTGYGGGGAGAAGCAA and primer R-TGTTGGTCCCAATTGTCTCCCCTC. The PCR products were ligated into the T vector according to the protocol of the pEASY-T3 Cloning Kit (Transgen, China) and then transformed into the Trans5α competent cells (CWBIO, China). Six bacterial clones of each sample were picked for sequencing. The sequencing results were analyzed on the IMGT/HLA (https://www.ebi.ac.uk/ipd/imgt/hla/) website^41^.

### PBMC isolation

Blood samples of COVID-19 recovered donors were obtained from Yongchuan Hospital of Chongqing Medical University. Additionally, blood samples of unexposed donors were obtained from Chongqing Medical University. All these blood samples were preserved in ethylene diamine tetra acetic acid (EDTA) tubes and isolated the PBMCs within 16 h. The PBMCs were isolated via density centrifugation (Ficoll-Paque) (GE). Isolated PBMCs were cryopreserved and stored in liquid nitrogen until used in the assays.

### In vitro stimulation of 4-1BB^+^ CD8^+^ T cells with K562 cell lines

To use the K562 cells as the antigen for T cell stimulation, the cells were pre-treated with 10 μg/ml Mitomycin C (Meilunbio, China) for 2 h in the 37 °C cell incubator. After washing 3 times with the PBS medium, three K562 expressing TMG1, TMG2, and TMG3 were mixed in a ratio of 1:1:1 and added to the PBMC culture medium without IL-2 following the ratio of PBMC: K562 = 30:1. On day 5 and day 7, appropriate pre-treated K562 cells were supplemented. On day 10, three K562 expressing TMG1, TMG2, and TMG3 were mixed in a ratio of 1:1:1 and added to the PBMC culture medium with 50 IU/mL IL-2 following the ratio of PBMC: K562 = 4:1. The cells were cultured in an incubator at 37 °C for 5 days (day 15). During the period, 1/2 fluid change was performed 1–2 times according to the cell proliferation status.

On day 15, we added the mixed K562 cells to stimulate the expanded PBMC cultured in the 1640 medium without IL-2 following the ratio of PBMC: K562 = 4:1. These cells were used for the flow cytometry analysis.

### Flow cytometry analysis

We collected 50ul cells on day 10 and day 16 after stimulation for flow cytometry analysis. We collected the cells by centrifuge at 1100 rpm for 5 min and stained the cell with the anti-human CD3-PB antibody (1:200, Biolegend, cat#317313), anti-human CD8-APC (1:200, Biolegend, cat#301014), anti-human CD4-FITC (1:200, Biolegend, cat#317407), and anit-4-1BB-PE (1:200, Biolegend, cat#309803) for 30 min at room temperature, washed with PBS + 1% FBS and read with the LSRFortessa flow cytometer. The data were analyzed by the FlowJ software (FlowJo v10). 4-1BB was used as a marker of T cell activation^42,43^.

For analyzing the CD69 expression after the co-culturing, 150 μl cells were stained with the anti-human CD69-APC antibody (1:200, Biolegend, cat#310910). Other operations were the same as described above.

### IFN-γ ELISpot assay

IFN-γ ELISpot assays were performed according to the protocol of the Human IFN-γ pre-coated ELISpot kit (Dakewe, cat#2110005). Briefly, the pre-coated IFN-γ ELISpot was active by the 1640 medium without FBS. Then 5 × 10^3^ T cells were seeded per well, and add 5 × 10^3^ mitomycin-treated K562 cells were added for stimulation. The T cells without K562 cells added were regarded as the negative control and the PHA as the positive control. After 20 h incubation at 37 °C with 5% CO_2_ as reported. Subsequently, the plates were developed with a human biotinylated IFN-γ detection antibody, followed by incubation with Streptavidin-HRP and AEC solution. The spots were qualified with the ELISpot Reader (AID, Germany).

In this experiment, K562 cells used for ELISpot on day 15 were different from those added into the co-culture medium from day 1 to day 10. For instance, “TMGmix-TMG2/3” means TMGmix K562 cells were used from day 1 to day 10, while K562 cells carrying TMG2 and TMG3 were used for ELISpot on day 15.

### Single-cell transcriptome and TCR sequencing

Each sample was stained with anti-CD8 (Biolegend, cat#301014), anti-CD4 (Biolegend, cat#317407), and anti-4-1BB (Biolegend, cat#309803) antibodies for 30 min at room temperature. DAPI was added before FACS. Single-cell isolation was performed using Aria III (BD). We sorted different T cell populations within the DAPI-CD3+ gate, including CD8^+^4-1BB^+^, CD8^+^ 4-1BB^−^, CD8^−^ 4-1BB^+^, CD8^−^ 4-1BB^−^, CD4^+^ 4-1BB^+^, CD4^+^4-1BB^−^, CD4^−^4-1BB^+^ and CD4^−^4-1BB^−^ populations. All populations of each sample were mixed according to Table S1, and loaded into one channel of the 10x Chromium single cell 5’ immune profiling kit (10x Genomics, Pleasanton) according to the manufacturer’s instructions. The loaded cell numbers were between 10,000 and 14,000. All the subsequent steps were performed following the standard manufacturer’s protocols. Gene expression and T cell receptor libraries were analyzed by an Illumina NovaSeq 6000 sequencer to generate 150-bp paired-end reads, following single-cell isolation, library preparation, and sequencing.

### Data processing of scRNA-seq

FASTQ files of 10x scRNA sequencing data were processed with GRCh38 reference genome using Cell Ranger (version 6.1.2, 10x Genomics). Different batches were merged, and cells with less than 400 UMI counts, less than 200 genes, or >10% of mitochondrial gene counts were removed. Genes detected in <10 cells were filtered out. The filtered expression matrix was normalized by the total number of UMIs per cell and was log2-transformed. Harmony^44^ was applied to the principal component analysis (PCA) components to remove batch effects for subsequent uniform manifold approximation and projection (UMAP) as well as clustering with the Leiden algorithm. T cells without the information of TCR β chains were filtered out. MAGIC^45^ was used to impute gene expression and denoise the count matrix for expression analysis.Transcriptome data was analyzed using BCL2fastq v2.20 (https://support.illumina.com/downloads/bcl2fastq-conversion-software-v2-20.html), Cell Ranger (https://support.10xgenomics.com/single-cell-gene-expression/software/release-notes/3-1, version 3.1), R (https://www.r-project.org/, version 4.0.1), R Studio (https://www.rstudio.com/), Python (https://www.python.org/,version 3.6.7), Scanpy(https://scanpy.readthedocs.io/en/stable/, version 1.8.2).

### VDJ-usage score

To examine the VDJ gene usage of TCRs in different clusters without the influence of imbalanced batch size of single-cell sequencing, we designed a voting scheme for analyzing BV, BJ, AV, and AJ gene usages separately: for a given cell type cluster, each batch voted for the top 3 VDJ genes. Then, the total votes of a specific VDJ gene in the given cluster would be the raw score. Finally, the raw scores for different VDJ genes in the same cluster were scaled to [0, 1] as the VDJ-usage score. For example, when analyzing the usage of TRBV genes, TRBV9 got the most votes from different batches in CD8_Activated and CD8_Central_Memory clusters, therefore the VDJ-usage scores of TRBV9 in these two clusters equaled 1 after scaling.

### Activation of Jurkat T cells by co-culture with K562 cells

For the peptide-loaded K562-1101 co-culture assay, we synthesized peptides, including AYKTFPPTEPKK, KTFPPTEPK, YKTFPPTEP, TFPPTEPKK (GenScript), with a purity of over 95%. Lyophilized peptides were reconstituted in dimethylsulfoxide to a concentration of 10 mM and subsequently diluted in water to create a 2 mM working stock. Finally, the peptides were diluted to 2 μM using a serum-free medium. In a 96-well U-bottom plate, 5 × 10^4^ K562-1101 cells were pulsed with 25 μl of the peptide solution and incubated for 2 h at 37 °C. After incubation, 100 μl of medium was added, followed by centrifugation at 1500 rpm for 5 min to remove the supernatant. The pellet was then washed once with 200 μl medium and finally resuspended in 100 μl medium. We mixed 25 × 10^4^ specific Jurkat cells and 5 × 10^4^ peptide-loaded-K562-1101 cells in the 96-well U-bottom plate and co-incubated for 6 h at 3 °C. After 6 h incubation, we collected the cells for the flow cytometry analysis.

For peptide-expressing K562 cells, we mixed 2 × 10^5^ Jurkat cells and 1 × 10^5^ K562 cells in the 48-well plate. After 7 h incubation, we collected the cells for the flow cytometry analysis.

### Cytokine release ELISA

To verify the function of SARS-CoV-2-specific TCR clones, we measured cytokine concentration in coculture mediums using enzyme-linked immunosorbent assays according to the manufacturer’s instructions (IL-2 Human Uncoated ELISA Kit, Thermos Fisher Scientific, Catalog # 88-7025-88). In detail, we coculture 1 × 10^5^ JC5(TCRα/β knockout Jurkat cell clone 5) cell line expressing single TCR clone with 1 × 10^5^ K562 target cells expressing matched single epitope for 20 h, We collect supernatant of coculture media for cytokine measurement.

### pMHC-displaying yeast and co-culture

The yeast strain (EBY100α) and plasmids employed for the integrated constitutive expression of pMHC were derived from the ysynalpha_Dest plasmid, kindly provided by Dr. Eric Klavins from the Electrical Engineering Department at the University of Washington, United States^46^. Peptides of varying lengths, originating from AYKTFPPTEPKK, were fused to HLA-A*11:01 molecules. The ysynalpha_Dest plasmid was linearized using NheI and XhoI restriction enzymes. The pMHC sequences were synthesized to include overlap regions compatible with the linearized plasmid. Subsequent cloning steps were carried out following the protocol of the 2× Seamless Cloning Kit from Beyotime Biotechnology, Catalog # D7010M. The yeast strains were constructed using the Conventional lithium acetate conversion method using ∼500 ng of plasmid digested with PmeI. For co-culture, in a 12-well plate, we mixed 1 × 10^5^ targeted T cells with appropriate yeast cells according to the E:T ratio and cultured them in RPMI 1640 medium (Gibco, #11875093) supplemented with 10% (v/v) FBS at 37 °C and 5% CO_2_. After 20 h of co-culturing, the cells were stained with anti-CD69-APC (1:200, BioLegend, #310910) and analyzed using an LSRFortessa flow cytometer.

### Statistics and reproducibility

Statistical analyses were performed with the Scipy 1.6.2 library in Python. The type of statistical tests, sample sizes, and number of replicates are provided in the corresponding figure legend. *p* < 0.05 was considered statistically significant. *p < 0.05, **p < 0.01, ***p < 0.001, and ****p < 0.0001. For experiments using cell lines, three biological replicates were used. For experiments using donors’ cells, the number of biological replicates was defined by the number of available samples and the number of technical replicates was defined by the number of cells within each sample.

### Reporting summary

Further information on research design is available in the Nature Portfolio Reporting Summary linked to this article.

## Supplementary information

Supplementary Information

Description of Additional Supplementary Files

supplementary Data 1

supplementary Data 2

supplementary Data 3

supplementary Data 4

Supplementary Data 5

Reporting Summary

## Data Availability

The sequences of newly generated plasmids were available in supplementary information. The processed expression matrix, TCR information, and cell annotations in this paper are available in the OMIX database with accession ID: OMIX001069, https://ngdc.cncb.ac.cn/omix/release/OMIX001069. The raw data of single-cell sequencing have been deposited into the GSA-human database with accession ID: HRA002230, https://ngdc.cncb.ac.cn/gsa-human/browse/HRA002230. All source data for the figures were provided as Supplementary Data 5 with this paper. All other data are available from the corresponding author upon reasonable request.
