# Supplementary material for: Identification of virus epitopes and reactive T-cell receptors from memory T cells without peptide synthesis
Source: Commun Biol. 2024 Nov 4;7:1432. doi: 10.1038/s42003-024-07048-x (PMC11535475; doi:10.1038/s42003-024-07048-x)
Supplement: Supplementary file 1 — Supplementary Information [file 42003_2024_7048_MOESM1_ESM.pdf]

Supplementary Information

Information in this file:

Table S1: Sorting strategies for single-cell sequencing
Figure S1: Analysis of the peptidomes of coronaviruses.
Figure S2: Gating strategy of fluorescence-activated cell sorting (FACS) and FACS for T cells from unexposed donors.
Figure S3: FACS for T cells from recovered donors.
Figure S4: Results of enzyme-linked immune absorbent spot (ELISpot) assays and barplots of FACS results.
Figure S5: Additional characteristics of TCRs.
Figure S6: FACS gating strategy of the pMHC-displaying yeast system and the peptide pulsing experiments.
Plasmid sequences

Information in separated files:
Supplementary Data 1: SARS-CoV-2 peptidome and presentation predictions for 139 HLA alleles.
Supplementary Data 2: SARS-CoV-2 sequences included in TMGs.
Supplementary Data 3: Donor information.
Supplementary Data 4: TCR sequences of engineered Jurkat T cells.

Table S1. Sorting strategies for single-cell sequencing

Batch	41BB$^+$ CD4$^+$	41BB$^+$ CD8$^+$	41BB$^-$ CD8$^+$	41BB$^-$ CD4$^+$
V7_M_Q2	0%	100%	0%	0%
V7_M_other	33.3%	0%	33.3%	33.3%
V7_NC	25%	25%	25%	25%
V10_M	10%	78%	10%	2%
V13_M	10%	78%	10%	2%
V14_M	10%	78%	10%	2%
V15_M	10%	78%	10%	2%
V16_V17_M	10%	78%	10%	2%
V20_M	10%	78%	10%	2%
NC1	10%	78%	10%	2%
NC2	10%	78%	10%	2%

Due to the low amount of 41BB$^+$CD8$^+$ T cells in negative control groups, V10_NC, V14_NC and V16_NC were mixed after FACS to get NC1; V13_NC, V15_NC, V17_NC and V20_NC were mixed for NC2. Besides, V16_M and V17_M were mixed to get V16_V17_M, also because of the low amount of cells. "M“ indicates cells co-cultured with K562 cells expressing TMGmix (TMG1, TMG2, and TMG3). “NC” indicates cells co-cultured with K562 cells expressing no TMG.

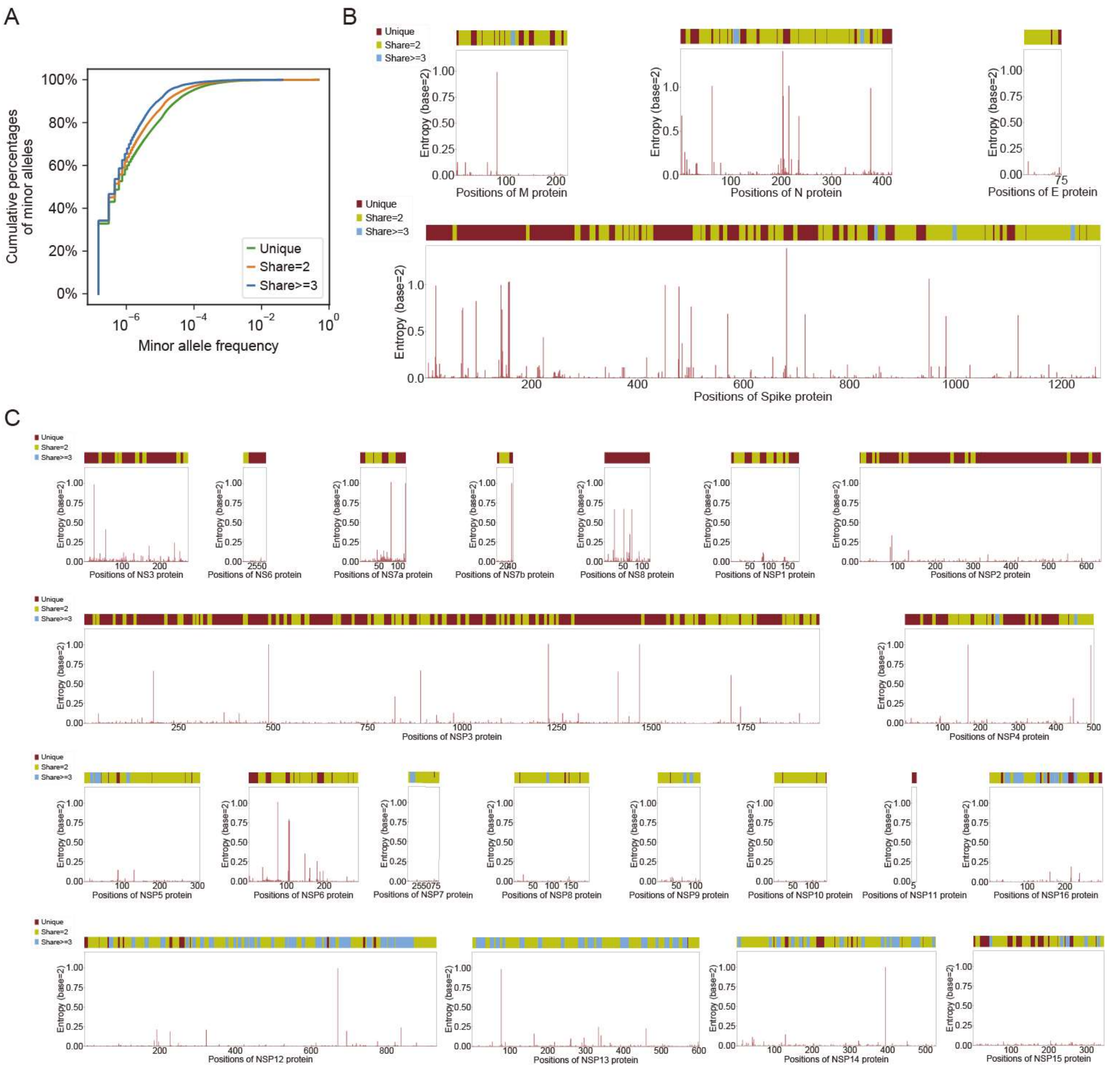

Figure S1: Analysis of the peptidomes of coronaviruses.

A. Cumulative curves showing the distribution of minor allele frequency of amino acids. Minor allele frequency tends to be low in conservative regions. A minor allele of an amino acid site was defined as any amino acid other than the most prevalent amino acid. A low minor allele frequency is expected due to negative selection if a site is conserved.

B. The entropy distribution along the Spike, N, E, and M proteins of SARS-CoV-2. The bar above indicates the group of sites.

C. The entropy distribution along non-structural proteins (NSPs).

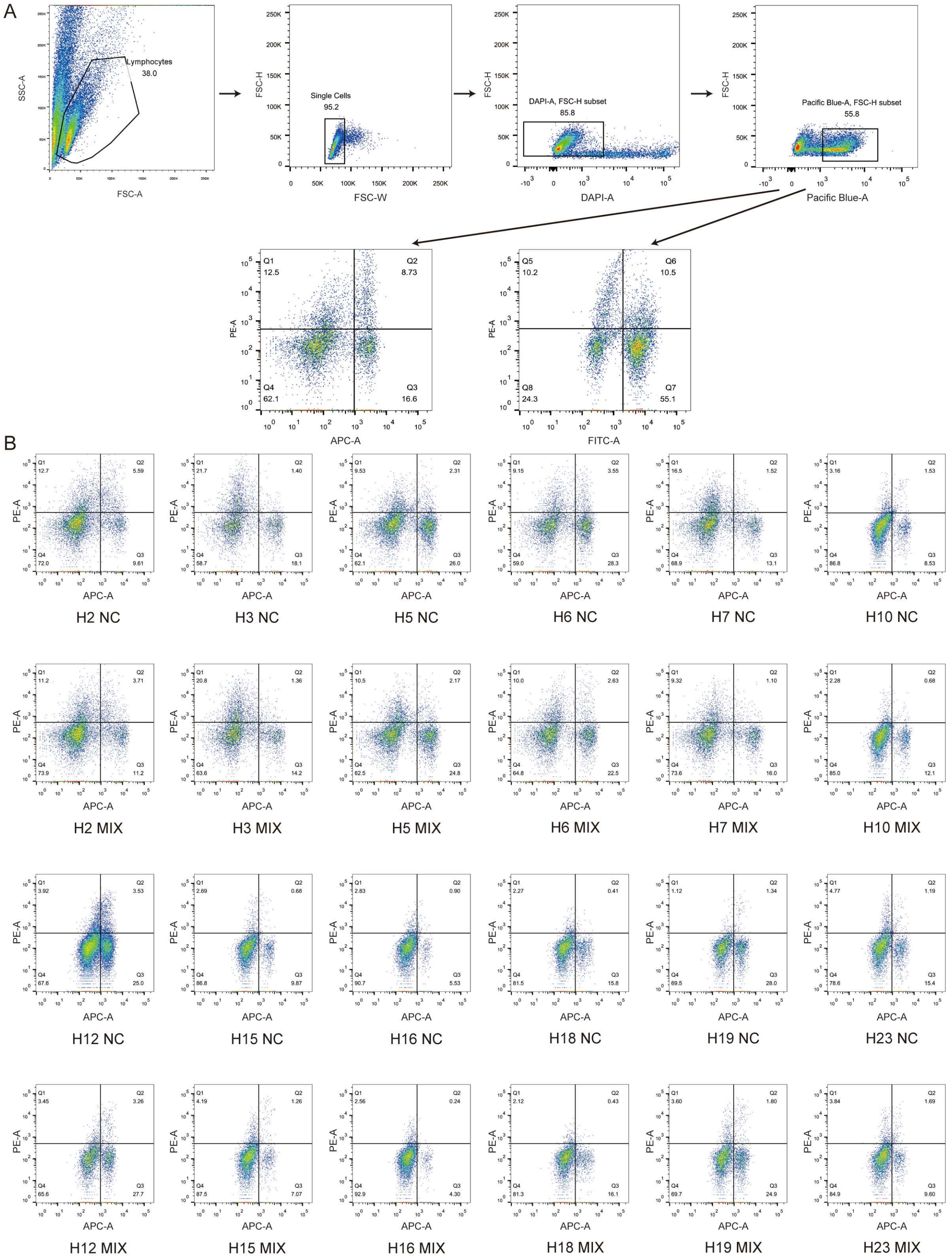

Figure S2. Gating strategy of fluorescence-activated cell sorting (FACS) and FACS for T cells from unexposed donors. A. FACS gating strategy of T cells. **B.** Q1-Q4 of FACS results of T cells from unexposed donors. Pacific Blue: anti-human CD3 antibody; APC: anti-human CD8a antibody; FITC: anti-human CD4 antibody; PE: anti-human CD137 (4-1BB) antibody.

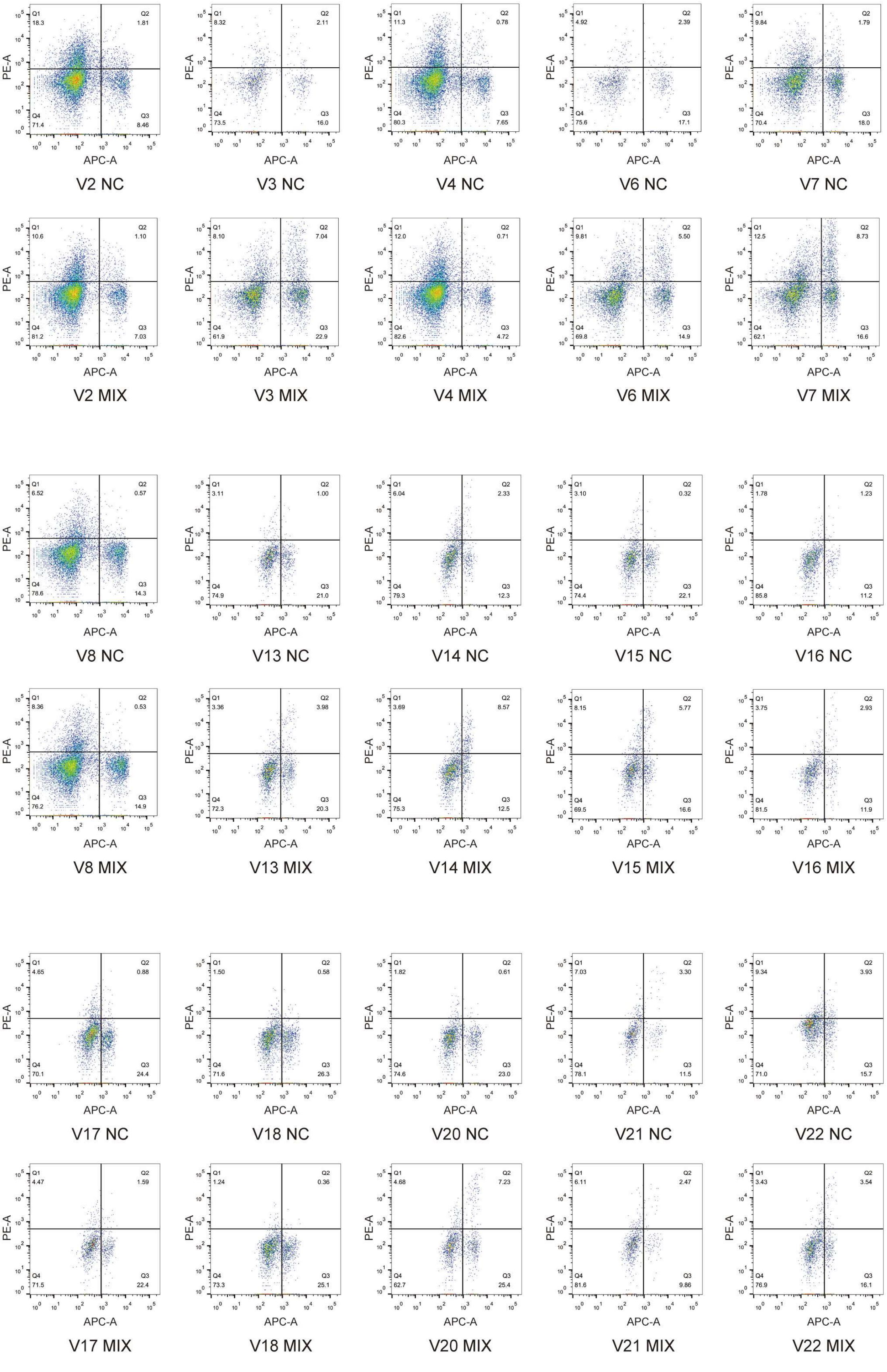

Figure S3. FACS for T cells from recovered donors. APC: anti-human CD8a antibody; PE: anti-human CD137 (4-1BB) antibody.

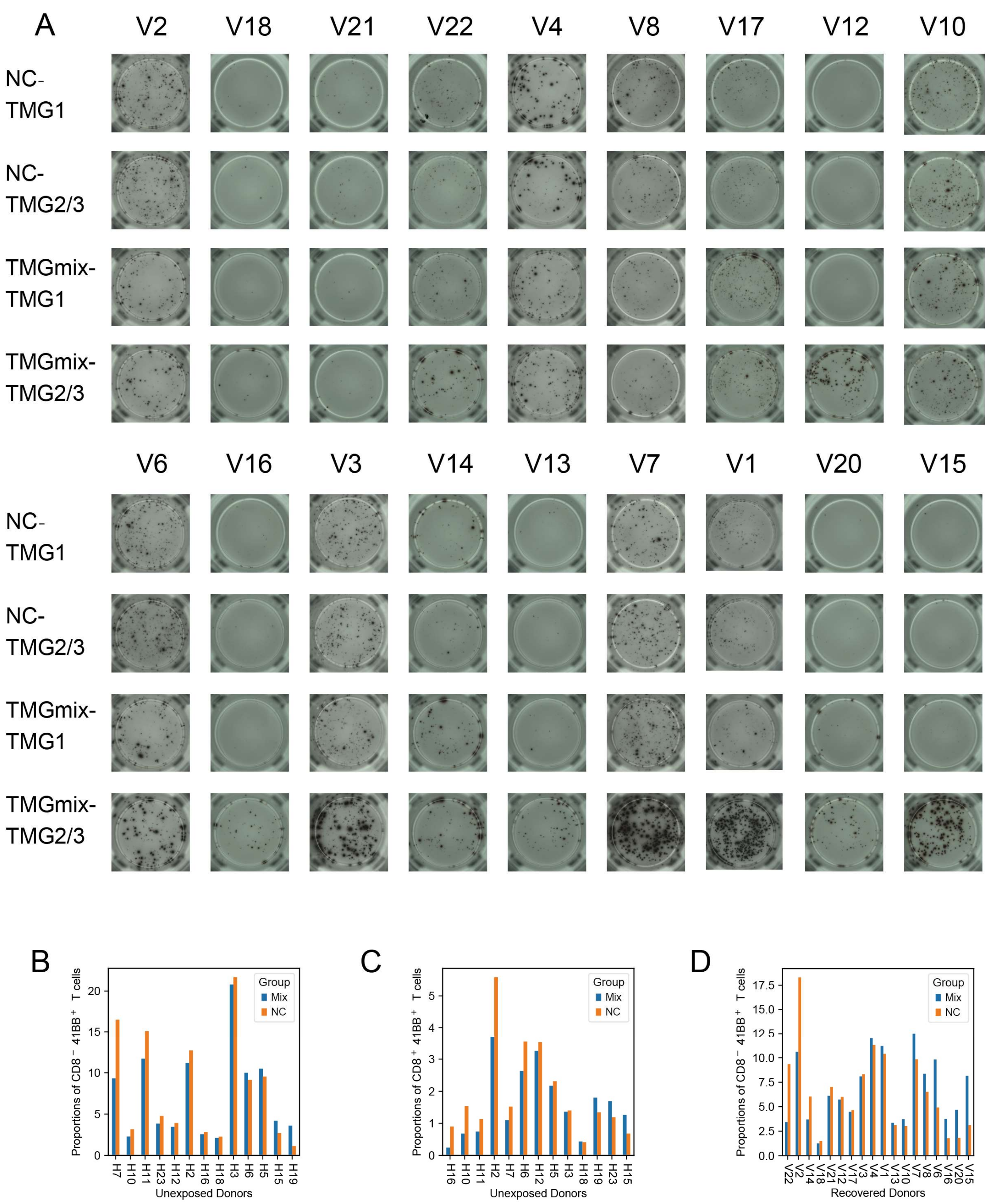

Figure S4. Results of enzyme-linked immune absorbent spot (ELISpot) assays and barplots of FACS results.

A. Results of enzyme-linked immune absorbent spot (ELISpot) assays. Labels on the left indicate the K562 cell groups. For instance, "TMGmix-TMG2/3" means TMGmix K562 cells were used for coculture from day 1 to day 10, while K562 cells carrying TMG2 and TMG3 were used for ELISpot on day 15. NC: negative control. TMGmix: K562 cells carrying TMG1, TMG2 and TMG3.

B. Barplot of the FACS result of $CD8^{-}$ $41BB^{+}$ T cells from unexposed donors.

C. Barplot of the FACS result of $CD8^{+}$ $41BB^{+}$ T cells from unexposed donors.

D. Barplot of the FACS result of $CD8^{-}$ $41BB^{+}$ T cells from recovered donors.

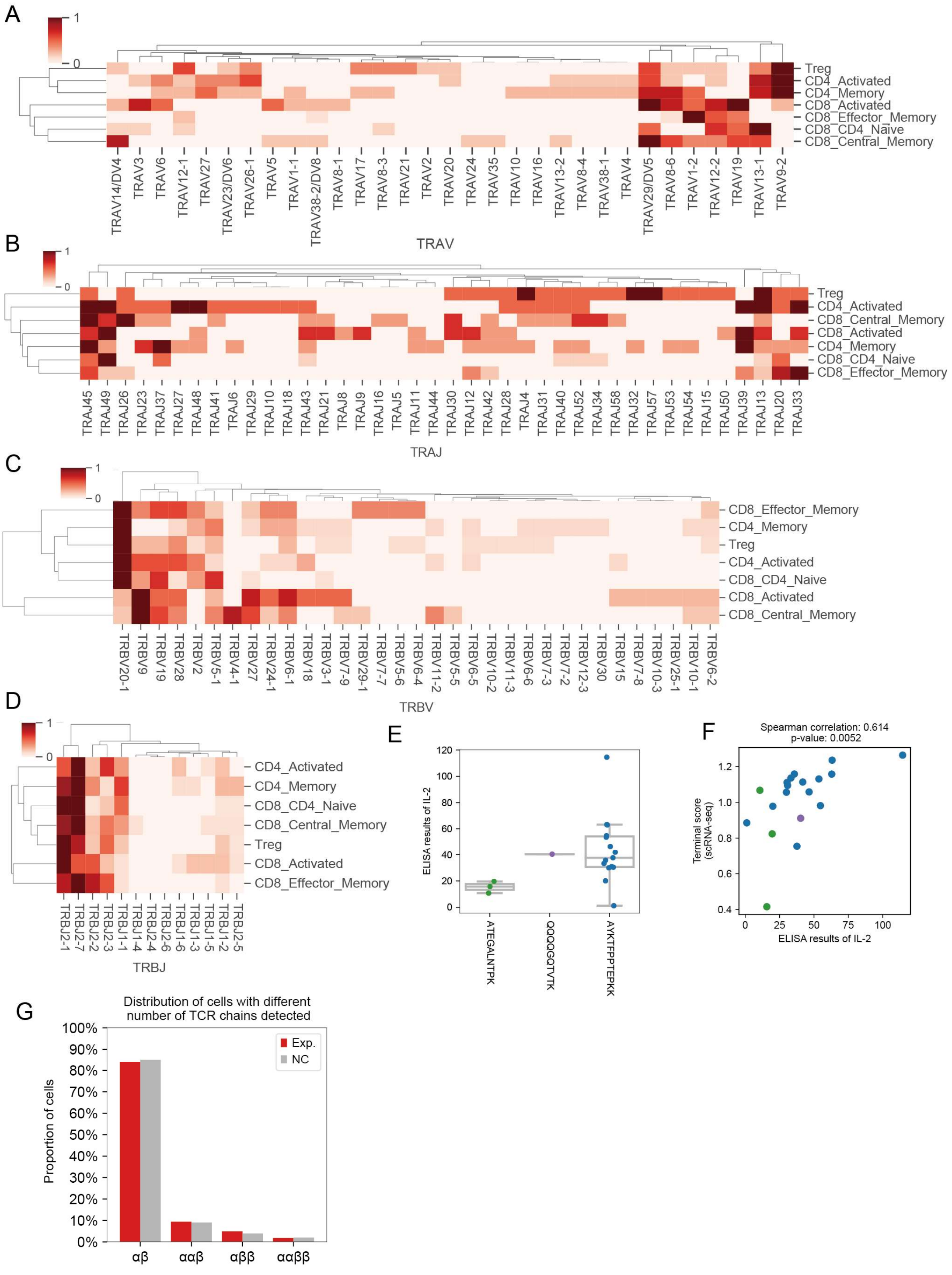

Figure S5: Additional characteristics of TCRs.

A-D. VDJ usage scores of TRAV (A), TRAJ (B), TRBV (C) and TRBJ (D) genes.

E. ELISA results of IL-2 released by Jurkat T cells with different TCRs targeting different epitopes. Each dot represents the mean value of T cells with a specific TCR.

F. Scatter plot showing the correlation between ELISA results of IL-2 and terminal scores of cells. Each dot represents the mean value of T cells with a specific TCR.

G. Distributions of cells with different numbers of α and β chains in the experimental group (Exp.) and the negative control group (NC).

A **AYKTFPPTEPKK-HLA-A*1101 displaying Yeast co-culture with TCR-01-1 Jurkat cells**

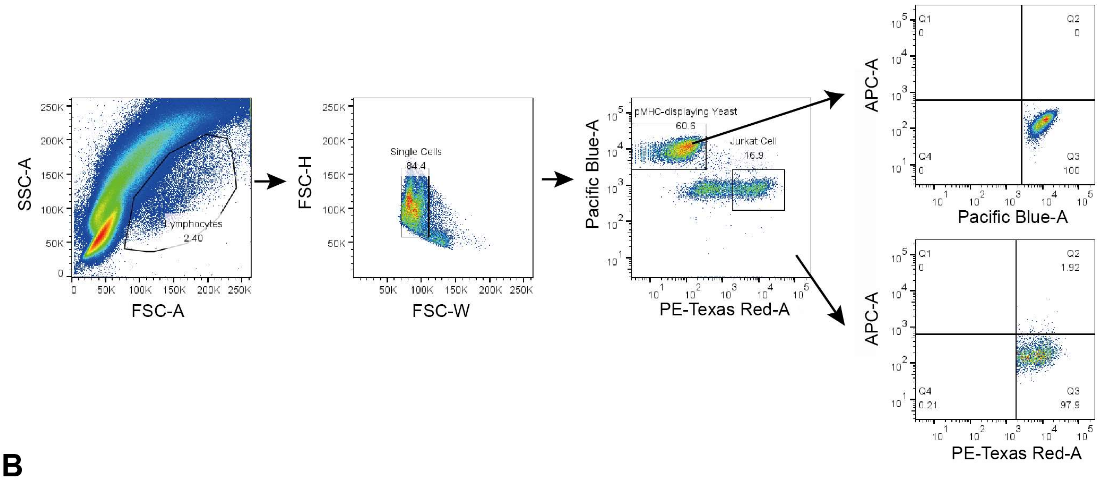

B

KTFPPTEPK-HLA-A*1101 displaying Yeast co-culture with TCR-01-1 Jurkat cells

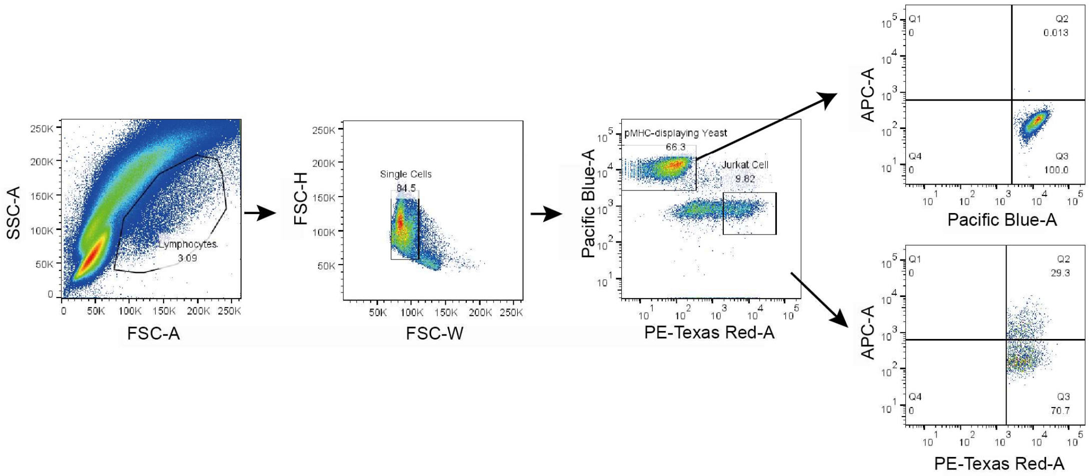

C

AYKTFPPTEPKK-loaded K562-1101 cell co-culture with TCR-01-1 Jurkat cells

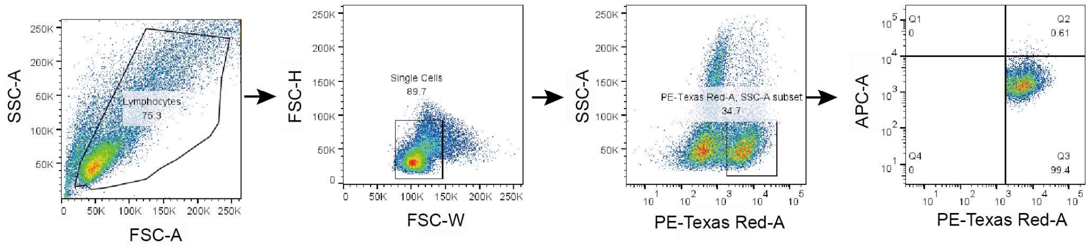

KTFPPTEPK-loaded K562-1101 cell co-culture with TCR-01-1 Jurkat cells

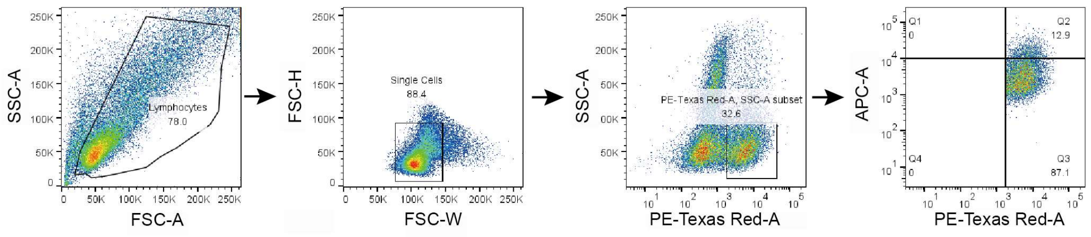

Figure S6: FACS gating strategy of the pMHC-displaying yeast system and the peptide pulsing experiments.

A-B. Representative results of flow cytometry analysis for AYKTFPPTEPKK-HLA-A*1101 displaying yeasts (**A**) and KTFPPTEPK-HLA-A*1101 displaying yeasts (**B**) co-cultured with TCR-01-1 Jurkat cells. The percentage of CD69+ T cells was calculated by first gating the RFP-expressing Jurkat cells, and then determining the proportion of activated T cells of the total T cell population. Pacific Blue: mTurquoise-expressing pMHC-displaying yeast.
C. Representative results of flow cytometry analysis for peptide-loaded K562-1101 co-cultured with TCR-01-1 Jurkat cells. APC: anti-human CD69 antibody; PE-Texas Red: RFP-expressing Jurkat cells. The same gating strategy was used for Fig. 5A-C.

Plasmid sequences

TMG1 (for K562)
tggaagggctaattcactcccaaagaagacaagatatccttgatctgtggatctaccacacacaaggctacttccctga
ttagcagaactacacaccagggccaggggtcagatatccactgacctttggatggtgctacaagctagtaccagttga
gccagataaggtagaagaggccaataaaggagagaacaccagcttgttacaccctgtgagcctgcatgggatgga
tgacccggagagagaagtgttagagtggaggtttgacagccgcctagcatttcatcacgtggcccgagagctgcatc
cggagtacttcaagaactgctgatatcgagcttgctacaagggactttccgctggggactttccagggaggcgtggcct
gggcgggactggggagtggcgagccctcagatcctgcatataagcagctgctttttgcctgtactgggtctctctggtta
gaccagatctgagcctgggagctctctggctaactagggaacccactgcttaagcctcaataaagcttgccttgagtgc
ttcaagtagtgtgtgcccgtctgttgtgtgactctggtaactagagatccctcagacccttttagtcagtgtggaaaatctct
agcagtggcgcccgaacagggacttgaaagcgaaagggaaaccagaggagctctctcgacgcaggactcggctt
gctgaagcgcgcacggcaagaggcgaggggcggcgactggtgagtacgccaaaaattttgactagcggaggcta
gaaggagagagatgggtgcgagagcgtcagtattaagcgggggagaattagatcgcgatgggaaaaaattcggtt
aaggccagggggaaagaaaaaatataaattaaaacatatagtatgggcaagcagggagctagaacgattcgcag
ttaatcctggcctgttagaaacatcagaaggctgtagacaaatactgggacagctacaaccatcccttcagacaggat
cagaagaacttagatcattatataatacagtagcaaccctctattgtgtgcatcaaaggatagagataaaagacacca
aggaagctttagacaagatagaggaagagcaaaacaaaagtaagaccaccgcacagcaagcggccggccgct
gatcttcagacctggaggaggagatatgagggacaattggagaagtgaattatataaatataaagtagtaaaaattg
aaccattaggagtagcacccaccaaggcaaagagaagagtggtgcagagagaaaaaagagcagtgggaatag
gagctttgttccttgggttcttgggagcagcaggaagcactatgggcgcagcgtcaatgacgctgacggtacaggcca
gacaattattgtctggtatagtgcagcagcagaacaatttgctgagggctattgaggcgcaacagcatctgttgcaactc
acagtctggggcatcaagcagctccaggcaagaatcctggctgtggaaagatacctaaaggatcaacagctcctgg
ggatttggggttgctctggaaaactcatttgcaccactgctgtgccttggaatgctagttggagtaataaatctctggaac
agatttggaatcacacgacctggatggagtgggacagagaaattaacaattacacaagcttaatacactccttaattg
aagaatcgcaaaaccagcaagaaaagaatgaacaagaattattggaattagataaatgggcaagtttgtggaattg
gtttaacataacaaattggctgtggtatataaaattattcataatgatagtaggaggcttggtaggtttaagaatagtttttgc
tgtactttctatagtgaatagagttaggcagggatattcaccattatcgtttcagacccacctcccaaccccgaggggac
ccgacaggcccgaaggaatagaagaagaaggtggagagagagacagagacagatccattcgattagtgaacgg
atctcgacggtatcgccgaattcacaaatggcagtattcatccacaattttaaaagaaaaggggggattggggggtac
agtgcaggggaaagaatagtagacataatagcaacagacatacaaactaaagaattacaaaaacaaattacaaa
aattcaaaattttcgggtttattacagggacagcagagatccagtttggactagtcgtgaggctccggtgcccgtcagtg

ggcagagcgcacatcgcccacagtccccgagaagttgggggagggtcggcaattgaaccggtgcctagagaa
ggtggcgcggggtaaactgggaaagtgatgtcgtgtactggctccgcctttttcccgagggtgggggagaaccgtata
taagtgcagtagtcgccgtgaacgttctttttcgcaacgggtttgccgccagaacacaggtaagtgccgtgtgtggttcc
cgcgggcctggcctctttacgggttatggcccttgcgtgccttgaattacttccacgcccctggctgcagtacgtgattcttg
atcccgagcttcgggttggaagtgggtgggagagttcgaggccttgcgcttaaggagccccttcgcctcgtgcttgagtt
gaggcctggcctgggcgctggggccgccgcgtgcgaatctggtggcaccttcgcgcctgtctcgctgctttcgataagt
ctctagccatttaaaatttttgatgacctgctgcgacgctttttttctggcaagatagtcttgtaaatgcgggccaagatctgc
acactggtatttcggtttttggggccgcgggcggcgacggggcccgtgcgtcccagcgcacatgttcggcgaggcgg
ggcctgcgagcgcggccaccgagaatcggacgggggtagtctcaagctggccggcctgctctggtgcctggcctcg
cgccgccgtgtatcgccccgccctgggcggcaaggctggcccggtcggcaccagttgcgtgagcggaaagatggc
cgcttcccggccctgctgcagggagctcaaaatggaggacgcggcgctcgggagagcgggcgggtgagtcaccc
acacaaaggaaaagggcctttccgtcctcagccgtcgcttcatgtgactccacggagtaccgggcgccgtccaggca
cctcgattagttctcgagcttttggagtacgtcgtctttaggttgggggggaggggttttatgcgatggagtttccccacactg
agtgggtggagactgaagttaggccagcttggcacttgatgtaattctccttggaatttgccctttttgagtttggatcttggtt
cattctcaagcctcagacagtggttcaaagtttttttcttccatttcaggtgtcgtgaagcggccgcGCCACCATGG
ATGTAGAAAACCCTCACCTTATGGGTTGGGATTATCCTAAATGTGATAGAGCCAT
GAAACCAGGTGGAACCTCATCAGGAGATGCCACAACTGCTTATGCTAATAGTGT
TTTTAAGTCAGTTCTTTATTATCAAAACAATGTTTTTATGTCTGAAGCAAAATGTT
GGACTGAGACTAAAGGACCTCATGAATTTTGCTCTCAACATACAATGCTAGTTAA
ACAGGGTGATGATTATATTGAACGGTTCGTGTCTTTAGCTATAGATGCTTACCCA
CTTACTAAACATCCTAATCAGGAGACAGCTTGCTCTCATGCCGCTGTTGATGCA
CTATGTGAGAAGGCATTAAAATATTTGGCTGAAATTGTTGACACTGTGAGTGCTT
TGGTTTATGATAATAAGCTTAAAGCACATAAAGACAAAGTACCCTATAATATGAG
AGTTATACATTTTGGTGCTGGTTCTGATAAAGGAGTTGCACCAGGTTTTATACAA
CAAAAGCTAGCTCTTGGAGGTTCCGTGGCTATAAAGATAACAGAACATTCTACC
GGTTATCCGTACGACGTGCCTGATTATGCCTGAtctagacccccccccctaacgttactggccg
aagccgcttggaataaggccggtgtgcgtttgtctatatgttattttccaccatattgccgtcttttggcaatgtgagggccc
ggaaacctggccctgtcttcttgacgagcattcctaggggtctttcccctctcgccaaaggaatgcaaggtctgttgaatg
tcgtgaaggaagcagttcctctggaagcttcttgaagacaaacaacgtctgtagcgaccctttgcaggcagcggaac
cccccacctggcgacaggtgcctctgcggccaaaagccacgtgtataagatacacctgcaaaggcggcacaaccc
cagtgccacgttgtgagttggatagttgtggaaagagtcaaatggctctcctcaagcgtattcaacaaggggctgaag
gatgcccagaaggtaccccattgtatgggatctgatctggggcctcggtgcacatgctttacatgtgtttagtcgaggtta
aaaaaacgtctaggccccccgaaccacggggacgtggttttcctttgaaaaacacgataataggatccATGACC
GAGTACAAGCCCACGGTGCGCCTCGCCACCCGCGACGACGTCCCCAGGGCCG
TACGCACCCTCGCCGCCGCGTTCGCCGACTACCCCGCCACGCGCCACACCGT
CGATCCGGACCGCCACATCGAGCGGGTCACCGAGCTGCAAGAACTCTTCCTCA
CGCGCGTCGGGCTCGACATCGGCAAGGTGTGGGTCGCGGACGACGGCGCCG
CGGTGGCGGTCTGGACCACGCCGGAGAGCGTCGAAGCGGGGGCGGTGTTCG
CCGAGATCGGCCCGCGCATGGCCGAGTTGAGCGGTTCCCGGCTGGCCGCGCA
GCAACAGATGGAAGGCCTCCTGGCGCCGCACCGGCCCAAGGAGCCCGCGTGG
TTCCTGGCCACCGTCGGAGTCTCGCCCGACCACCAGGGCAAGGGTCTGGGCA
GCGCCGTCGTGCTCCCCGGAGTGGAGGCGGCCGAGCGCGCCGGGGTGCCCG
CCTTCCTGGAGACCTCCGCGCCCCGCAACCTCCCCTTCTACGAGCGGCTCGGC
TTCACCGTCACCGCCGACGTCGAGGTGCCCGAAGGACCGCGCACCTGGTGCA

TGACCCGCAAGCCCGGTGCCTGAgctagcatcgatagatcctaatcaacctctggattacaaaatttgt
gaaagattgactggtattcttaactatgttgctccttttacgctatgtggatacgctgctttaatgcctttgtatcatgctattgctt
cccgtatggctttcattttctcctccttgtataaatcctggttgctgtctctttatgaggagttgtggcccgttgtcaggcaacgt
ggcgtggtgtgcactgtgtttgctgacgcaacccccactggttggggcattgccaccacctgtcagctcctttccgggact
ttcgctttccccctccctattgccacggcggaactcatcgccgcctgccttgcccgctgctggacaggggctcggctgttg
ggcactgacaattccgtggtgttgtcggggaaatcatcgtcctttccttggctgctcgcctgtgttgccacctggattctgcg
cgggacgtccttctgctacgtcccttcggccctcaatccagcggaccttccttcccgcggcctgctgccggctctgcggc
ctcttccgcgtcttcgccttcgccctcagacgagtcggatctccctttgggccgcctccccgcctgagatcctttaagacc
aatgacttacaaggcagctgtagatcttagccactttttaaaagaaaaggggggactggaagggctaattcactccca
acgaagacaagatctgctttttgcttgtactgggtctctctggttagaccagatctgagcctgggagctctctggctaacta
gggaacccactgcttaagcctcaataaagcttgccttgagtgcttcaagtagtgtgtgcccgtctgttgtgtgactctggta
actagagatccctcagacccttttagtcagtgtggaaaatctctagcagtagtagttcatgtcatcttattattcagtatttata
acttgcaaagaaatgaatatcagagagtgagaggcccgggttaattaaggaaagggctagatcattcttgaagacg
aaagggcctcgtgatacgcctatttttataggttaatgtcatgataataatggtttcttagacgtcaggtggcacttttcggg
gaaatgtgcgcggaacccctatttgtttatttttctaaatacattcaaatatgtatccgctcatgagacaataaccctgataa
atgcttcaataatattgaaaaaggaagagtatgagtattcaacatttccgtgtcgcccttattcccttttttgcggcattttgcc
ttcctgtttttgctcacccagaaacgctggtgaaagtaaaagatgctgaagatcagttgggtgcacgagtgggttacatc
gaactggatctcaacagcggtaagatccttgagagttttcgccccgaagaacgttttccaatgatgagcacttttaaagtt
ctgctatgtggcgcggtattatcccgtgttgacgccgggcaagagcaactcggtcgccgcatacactattctcagaatg
acttggttgagtactcaccagtcacagaaaagcatcttacggatggcatgacagtaagagaattatgcagtgctgccat
aaccatgagtgataacactgcggccaacttacttctgacaacgatcggaggaccgaaggagctaaccgcttttttgca
caacatgggggatcatgtaactcgccttgatcgttgggaaccggagctgaatgaagccataccaaacgacgagcgt
gacaccacgatgcctgtagcaatggcaacaacgttgcgcaaactattaactggcgaactacttactctagcttcccgg
caacaattaatagactggatggaggcggataaagttgcaggaccacttctgcgctcggcccttccggctggctggttta
ttgctgataaatctggagccggtgagcgtgggtctcgcggtatcattgcagcactggggccagatggtaagccctccc
gtatcgtagttatctacacgacggggagtcaggcaactatggatgaacgaaatagacagatcgctgagataggtgcc
tcactgattaagcattggtaactgtcagaccaagtttactcatatatactttagattgatttaaaacttcatttttaatttaaaag
gatctaggtgaagatcctttttgataatctcatgaccaaaatcccttaacgtgagttttcgttccactgagcgtcagacccc
gtagaaaagatcaaaggatcttcttgagatcctttttttctgcgcgtaatctgctgcttgcaaacaaaaaaaccaccgcta
ccagcggtggtttgtttgccggatcaagagctaccaactctttttccgaaggtaactggcttcagcagagcgcagatacc
aaatactgttcttctagtgtagccgtagttaggccaccacttcaagaactctgtagcaccgcctacatacctcgctctgct
aatcctgttaccagtggctgctgccagtggcgataagtcgtgtcttaccgggttggactcaagacgatagttaccggata
aggcgcagcggtcgggctgaacggggggttcgtgcacacagcccagcttggagcgaacgacctacaccgaactg
agatacctacagcgtgagctatgagaaagcgccacgcttcccgaagggagaaaggcggacaggtatccggtaag
cggcagggtcggaacaggagagcgcacgagggagcttccagggggaaacgcctggtatctttatagtcctgtcggg
tttcgccacctctgacttgagcgtcgatttttgtgatgctcgtcaggggggcggagcctatggaaaaacgccagcaacg
cggcctttttacggttcctggccttttgctggccttttgctcacatgttctttcctgcgttatcccctgattctgtggataaccgtat
taccgcctttgagtgagctgataccgctcgccgcagccgaacgaccgagcgcagcgagtcagtgagcgaggaagc
ggaagagcgcccaatacgcaaaccgcctctccccgcgcgttggccgattcattaatgcagcaagctcatggctgact
aattttttttatttatgcagaggccgaggccgcctcggcctctgagctattccagaagtagtgaggaggcttttttggaggc
ctaggcttttgcaaaaagctccccgtggcacgacaggtttcccgactggaaagcgggcagtgagcgcaacgcaatta
atgtgagttagctcactcattaggcaccccaggctttacactttatgcttccggctcgtatgttgtgtggaattgtgagcgga
taacaatttcacacaggaaacagctatgacatgattacgaatttcacaaataaagcatttttttcactgcattctagttgtg
gtttgtccaaactcatcaatgtatcttatcatgtctggatcaactggataactcaagctaaccaaaatcatcccaaacttcc

caccccatacccctattaccactgccaattacctgtggtttcatttactctaaacctgtgattcctctgaattattttcattttaaa
gaaattgtatttgttaaatatgtactacaaacttagtagt

TMG2 (for K562)
tggaagggctaattcactcccaaagaagacaagatatccttgatctgtggatctaccacacacaaggctacttccctga
ttagcagaactacacaccagggccaggggtcagatatccactgacctttggatggtgctacaagctagtaccagttga
gccagataaggtagaagaggccaataaaggagagaacaccagcttgttacaccctgtgagcctgcatgggatgga
tgacccggagagagaagtgttagagtggaggtttgacagccgcctagcatttcatcacgtggcccgagagctgcatc
cggagtacttcaagaactgctgatatcgagcttgctacaagggactttccgctggggactttccagggaggcgtggcct
gggcgggactggggagtggcgagccctcagatcctgcatataagcagctgctttttgcctgtactgggtctctctggtta
gaccagatctgagcctgggagctctctggctaactagggaacccactgcttaagcctcaataaagcttgccttgagtgc
ttcaagtagtgtgtgcccgtctgttgtgtgactctggtaactagagatccctcagacccttttagtcagtgtggaaaatctct
agcagtggcgcccgaacagggacttgaaagcgaaagggaaaccagaggagctctctcgacgcaggactcggctt
gctgaagcgcgcacggcaagaggcgaggggcggcgactggtgagtacgccaaaaattttgactagcggaggcta
gaaggagagagatgggtgcgagagcgtcagtattaagcgggggagaattagatcgcgatgggaaaaaattcggtt
aaggccagggggaaagaaaaaatataaattaaaacatatagtatgggcaagcagggagctagaacgattcgcag
ttaatcctggcctgttagaaacatcagaaggctgtagacaaatactgggacagctacaaccatcccttcagacaggat
cagaagaacttagatcattatataatacagtagcaaccctctattgtgtgcatcaaaggatagagataaaagacacca
aggaagctttagacaagatagaggaagagcaaaacaaaagtaagaccaccgcacagcaagcggccggccgct
gatcttcagacctggaggaggagatatgagggacaattggagaagtgaattatataaatataaagtagtaaaaattg
aaccattaggagtagcacccaccaaggcaaagagaagagtggtgcagagagaaaaaagagcagtgggaatag
gagctttgttccttgggttcttgggagcagcaggaagcactatgggcgcagcgtcaatgacgctgacggtacaggcca
gacaattattgtctggtatagtgcagcagcagaacaatttgctgagggctattgaggcgcaacagcatctgttgcaactc
acagtctggggcatcaagcagctccaggcaagaatcctggctgtggaaagatacctaaaggatcaacagctcctgg
ggatttggggttgctctggaaaactcatttgcaccactgctgtgccttggaatgctagttggagtaataaatctctggaac
agatttggaatcacacgacctggatggagtgggacagagaaattaacaattacacaagcttaatacactccttaattg
aagaatcgcaaaaccagcaagaaaagaatgaacaagaattattggaattagataaatgggcaagtttgtggaattg
gtttaacataacaaattggctgtggtatataaaattattcataatgatagtaggaggcttggtaggtttaagaatagtttttgc
tgtactttctatagtgaatagagttaggcagggatattcaccattatcgtttcagacccacctcccaaccccgaggggac
ccgacaggcccgaaggaatagaagaagaaggtggagagagagacagagacagatccattcgattagtgaacgg
atctcgacggtatcgccgaattcacaaatggcagtattcatccacaattttaaaagaaaaggggggattggggggtac
agtgcaggggaaagaatagtagacataatagcaacagacatacaaactaaagaattacaaaaacaaattacaaa
aattcaaaattttcgggtttattacagggacagcagagatccagtttggactagtcgtgaggctccggtgcccgtcagtg
ggcagagcgcacatcgcccacagtccccgagaagttggggggaggggtcggcaattgaaccggtgcctagagaa
ggtggcgcggggtaaactgggaaagtgatgtcgtgtactggctccgcctttttcccgagggtgggggagaaccgtata
taagtgcagtagtcgccgtgaacgttctttttcgcaacgggtttgccgccagaacacaggtaagtgccgtgtgtggttcc
cgcgggcctggcctctttacgggttatggcccttgcgtgccttgaattacttccacgcccctggctgcagtacgtgattcttg
atcccgagcttcgggttggaagtgggtgggagagttcgaggccttgcgcttaaggagccccttcgcctcgtgcttgagtt
gaggcctggcctgggcgctggggccgccgcgtgcgaatctggtggcaccttcgcgcctgtctcgctgctttcgataagt
ctctagccatttaaaatttttgatgacctgctgcgacgctttttttctggcaagatagtcttgtaaatgcgggccaagatctgc
acactggtatttcggtttttggggccgcgggcggcgacggggcccgtgcgtcccagcgcacatgttcggcgaggcgg
ggcctgcgagcgcggccaccgagaatcggacgggggtagtctcaagctggccggcctgctctggtgcctggcctcg
cgccgccgtgtatcgccccgccctgggcggcaaggctggcccggtcggcaccagttgcgtgagcggaaagatggc

cgcttcccggccctgctgcagggagctcaaaatggaggacgcggcgctcgggagagcgggcgggtgagtcaccc
acacaaaggaaaagggcctttccgtcctcagccgtcgcttcatgtgactccacggagtaccgggcgccgtccaggca
cctcgattagttctcgagcttttggagtacgtcgtctttaggttggggggaggggttttatgcgatggagtttccccacactg
agtgggtggagactgaagttaggccagcttggcacttgatgtaattctccttggaatttgccctttttgagtttggatcttggtt
cattctcaagcctcagacagtggttcaaagtttttttcttccatttcaggtgtcgtgaagcggccgcGCCACCATGA
ATTCTAGAATTAAAGCATCTATGCCGACTACTATAGCAAAGAATACTGTTAAGAG
TGCTATGGTTAGAATGTACATCTTCTTTGCATCATTTTATTATGTATGGAAAAGTT
ATCCACCTTGTAGGTTTGTTACAGACACACCTAAAGGTCCTAAAGTGAAGTATTT
ATACTTATTCTCTACAGTGTTCCCACCTACAAGTTTTGGACCACTAGTGAGAAAA
ATATTTAAATGGGGTAAGGCTAGACTTTATTATGATTCAATGAGTTATGAGGATC
AAGATGCAGCCGCCACTAGAGGAGCTACTGTAGTAATTGGAACAAGCAAATTCT
ATGGTGGTTGGTGTACTGAAAGACTCAAGCTTTTTGCAGCAGAAACGCTCAAAG
CTACTGAGGAGACAGACTATGGTGATGCTGTTGTTTACCGAGGTACAACAACTT
ACAAATTAAATGTTGGTATCTCAGATGAGTTTTCTAGCAATGTTGCAAATTATCAA
AAGGTTGGTATGCAAAAGCAAGCGTGGCAACCGGGTGTTGCTATGCCTAATCTT
TACAAAATGCAAAGAATGCTAACCGGTTATCCGTACGACGTGCCTGATTATGCC
TGAtctagacccccccccctaacgttactggccgaagccgcttggaataaggccggtgtgcgtttgtctatatgttatttt
ccaccatattgccgtcttttggcaatgtgagggcccggaaacctggccctgtcttcttgacgagcattcctaggggtctttc
ccctctcgccaaaggaatgcaaggtctgttgaatgtcgtgaaggaagcagttcctctggaagcttcttgaagacaaac
aacgtctgtagcgaccctttgcaggcagcggaaccccccacctggcgacaggtgcctctgcggccaaaagccacgt
gtataagatacacctgcaaaggcggcacaaccccagtgccacgttgtgagttggatagttgtggaaagagtcaaatg
gctctcctcaagcgtattcaacaaggggctgaaggatgcccagaaggtaccccattgtatgggatctgatctggggcc
tcggtgcacatgctttacatgtgtttagtcgaggttaaaaaaacgtctaggccccccgaaccacggggacgtggttttcc
tttgaaaaacacgataataggatccATGACCGAGTACAAGCCCACGGTGCGCCTCGCCACC
CGCGACGACGTCCCCAGGGCCGTACGCACCCTCGCCGCCGCGTTCGCCGACT
ACCCCGCCACGCGCCACACCGTCGATCCGGACCGCCACATCGAGCGGGTCAC
CGAGCTGCAAGAACTCTTCCTCACGCGCGTCGGGCTCGACATCGGCAAGGTGT
GGGTCGCGGACGACGGCGCCGCGGTGGCGGTCTGGACCACGCCGGAGAGCG
TCGAAGCGGGGGCGGTGTTCGCCGAGATCGGCCCGCGCATGGCCGAGTTGAG
CGGTTCCCGGCTGGCCGCGCAGCAACAGATGGAAGGCCTCCTGGCGCCGCAC
CGGCCCAAGGAGCCCGCGTGGTTCCTGGCCACCGTCGGAGTCTCGCCCGACC
ACCAGGGCAAGGGTCTGGGCAGCGCCGTCGTGCTCCCCGGAGTGGAGGCGG
CCGAGCGCGCCGGGGTGCCCGCCTTCCTGGAGACCTCCGCGCCCCGCAACCT
CCCCTTCTACGAGCGGCTCGGCTTCACCGTCACCGCCGACGTCGAGGTGCCC
GAAGGACCGCGCACCTGGTGCATGACCCGCAAGCCCGGTGCCTGAgctagcatcga
tagatcctaatcaacctctggattacaaaatttgtgaaagattgactggtattcttaactatgttgctccttttacgctatgtgg
atacgctgctttaatgcctttgtatcatgctattgcttcccgtatggctttcattttctcctccttgtataaatcctggttgctgtctct
ttatgaggagttgtggcccgttgtcaggcaacgtggcgtggtgtgcactgtgtttgctgacgcaacccccactggttggg
gcattgccaccacctgtcagctcctttccgggactttcgctttccccctccctattgccacggcggaactcatcgccgcct
gccttgcccgctgctggacaggggctcggctgttgggcactgacaattccgtggtgttgtcggggaaatcatcgtcctttc
cttggctgctcgcctgtgttgccacctggattctgcgcgggacgtccttctgctacgtcccttcggccctcaatccagcgg
accttccttcccgcggcctgctgccggctctgcggcctcttccgcgtcttcgccttcgccctcagacgagtcggatctccct
ttgggccgcctccccgcctgagatcctttaagaccaatgacttacaaggcagctgtagatcttagccactttttaaaaga
aaaggggggactggaagggctaattcactcccaacgaagacaagatctgctttttgcttgtactgggtctctctggttag

accagatctgagcctgggagctctctggctaactagggaacccactgcttaagcctcaataaagcttgccttgagtgctt
caagtagtgtgtgcccgtctgttgtgtgactctggtaactagagatccctcagacccttttagtcagtgtggaaaatctcta
gcagtagtagttcatgtcatcttattattcagtatttataacttgcaaagaaatgaatatcagagagtgagaggcccgggtt
aattaaggaaagggctagatcattcttgaagacgaaagggcctcgtgatacgcctatttttataggttaatgtcatgataa
taatggtttcttagacgtcaggtggcacttttcggggaaatgtgcgcggaacccctatttgtttatttttctaaatacattcaa
atatgtatccgctcatgagacaataaccctgataaatgcttcaataatattgaaaaaggaagagtatgagtattcaacat
ttccgtgtcgcccttattcccttttttgcggcattttgccttcctgtttttgctcacccagaaacgctggtgaaagtaaaagatg
ctgaagatcagttgggtgcacgagtgggttacatcgaactggatctcaacagcggtaagatccttgagagttttcgccc
cgaagaacgttttccaatgatgagcacttttaaagttctgctatgtggcgcggtattatcccgtgttgacgccgggcaaga
gcaactcggtcgccgcatacactattctcagaatgacttggttgagtactcaccagtcacagaaaagcatcttacggat
ggcatgacagtaagagaattatgcagtgctgccataaccatgagtgataacactgcggccaacttacttctgacaacg
atcggaggaccgaaggagctaaccgcttttttgcacaacatgggggatcatgtaactcgccttgatcgttgggaaccg
gagctgaatgaagccataccaaacgacgagcgtgacaccacgatgcctgtagcaatggcaacaacgttgcgcaa
actattaactggcgaactacttactctagcttcccggcaacaattaatagactggatggaggcggataaagttgcagga
ccacttctgcgctcggcccttccggctggctggtttattgctgataaatctggagccggtgagcgtgggtctcgcggtatc
attgcagcactggggccagatggtaagccctcccgtatcgtagttatctacacgacggggagtcaggcaactatggat
gaacgaaatagacagatcgctgagataggtgcctcactgattaagcattggtaactgtcagaccaagtttactcatata
tactttagattgatttaaaacttcatttttaatttaaaaggatctaggtgaagatcctttttgataatctcatgaccaaaatccct
taacgtgagttttcgttccactgagcgtcagaccccgtagaaaagatcaaaggatcttcttgagatcctttttttctgcgcgt
aatctgctgcttgcaaacaaaaaaaccaccgctaccagcggtggtttgtttgccggatcaagagctaccaactctttttc
cgaaggtaactggcttcagcagagcgcagataccaaatactgttcttctagtgtagccgtagttaggccaccacttcaa
gaactctgtagcaccgcctacatacctcgctctgctaatcctgttaccagtggctgctgccagtggcgataagtcgtgtct
taccgggttggactcaagacgatagttaccggataaggcgcagcggtcgggctgaacggggggttcgtgcacacag
cccagcttggagcgaacgacctacaccgaactgagatacctacagcgtgagctatgagaaagcgccacgcttccc
gaagggagaaaggcggacaggtatccggtaagcggcagggtcggaacaggagagcgcacgagggagcttcca
gggggaaacgcctggtatctttatagtcctgtcgggtttcgccacctctgacttgagcgtcgatttttgtgatgctcgtcagg
ggggcggagcctatggaaaaacgccagcaacgcggcctttttacggttcctggccttttgctggccttttgctcacatgtt
ctttcctgcgttatcccctgattctgtggataaccgtattaccgcctttgagtgagctgataccgctcgccgcagccgaacg
accgagcgcagcgagtcagtgagcgaggaagcggaagagcgcccaatacgcaaaccgcctctccccgcgcgtt
ggccgattcattaatgcagcaagctcatggctgactaattttttttatttatgcagaggccgaggccgcctcggcctctga
gctattccagaagtagtgaggaggcttttttggaggcctaggcttttgcaaaaagctccccgtggcacgacaggtttccc
gactggaaagcgggcagtgagcgcaacgcaattaatgtgagttagctcactcattaggcaccccaggctttacacttt
atgcttccggctcgtatgttgtgtggaattgtgagcggataacaatttcacacaggaaacagctatgacatgattacgaa
tttcacaaataaagcatttttttcactgcattctagttgtggtttgtccaaactcatcaatgtatcttatcatgtctggatcaactg
gataactcaagctaaccaaaatcatcccaaacttcccaccccataccctattaccactgccaattacctgtggtttcattt
actctaaacctgtgattcctctgaattattttcattttaaagaaattgtatttgttaaatatgtactacaaacttagtagt

TMG3 (for K562)
tggaagggctaattcactcccaaagaagacaagatatccttgatctgtggatctaccacacacaaggctacttccctga
ttagcagaactacacaccagggccaggggtcagatatccactgacctttggatggtgctacaagctagtaccagttga
gccagataaggtagaagaggccaataaaggagagaacaccagcttgttacaccctgtgagcctgcatgggatgga
tgacccggagagagaagtgttagagtggaggtttgacagccgcctagcatttcatcacgtggcccgagagctgcatc
cggagtacttcaagaactgctgatatcgagcttgctacaagggactttccgctggggactttccagggaggcgtggcct

gggcgggactggggagtggcgagccctcagatcctgcatataagcagctgctttttgcctgtactgggtctctctggtta
gaccagatctgagcctgggagctctctggctaactagggaacccactgcttaagcctcaataaagcttgccttgagtgc
ttcaagtagtgtgtgcccgtctgttgtgtgactctggtaactagagatccctcagacccttttagtcagtgtggaaaatctct
agcagtggcgcccgaacagggacttgaaagcgaaagggaaaccagaggagctctctcgacgcaggactcggctt
gctgaagcgcgcacggcaagaggcgaggggcggcgactggtgagtacgccaaaaattttgactagcggaggcta
gaaggagagagatgggtgcgagagcgtcagtattaagcgggggagaattagatcgcgatgggaaaaaattcggtt
aaggccagggggaaagaaaaaatataaattaaaacatatagtatgggcaagcagggagctagaacgattcgcag
ttaatcctggcctgttagaaacatcagaaggctgtagacaaatactgggacagctacaaccatcccttcagacaggat
cagaagaacttagatcattatataatacagtagcaaccctctattgtgtgcatcaaaggatagagataaaagacacca
aggaagctttagacaagatagaggaagagcaaaacaaaagtaagaccaccgcacagcaagcggccggccgct
gatcttcagacctggaggaggagatatgagggacaattggagaagtgaattatataaatataaagtagtaaaaattg
aaccattaggagtagcacccaccaaggcaaagagaagagtggtgcagagagaaaaaagagcagtgggaatag
gagctttgttccttgggttcttgggagcagcaggaagcactatgggcgcagcgtcaatgacgctgacggtacaggcca
gacaattattgtctggtatagtgcagcagcagaacaatttgctgagggctattgaggcgcaacagcatctgttgcaactc
acagtctggggcatcaagcagctccaggcaagaatcctggctgtggaaagatacctaaaggatcaacagctcctgg
ggatttggggttgctctggaaaactcatttgcaccactgctgtgccttggaatgctagttggagtaataaatctctggaac
agatttggaatcacacgacctggatggagtgggacagagaaattaacaattacacaagcttaatacactccttaattg
aagaatcgcaaaaccagcaagaaaagaatgaacaagaattattggaattagataaatgggcaagtttgtggaattg
gtttaacataacaaattggctgtggtatataaaattattcataatgatagtaggaggcttggtaggtttaagaatagtttttgc
tgtactttctatagtgaatagagttaggcagggatattcaccattatcgtttcagacccacctcccaaccccgaggggac
ccgacaggcccgaaggaatagaagaagaaggtggagagagagacagagacagatccattcgattagtgaacgg
atctcgacggtatcgccgaattcacaaatggcagtattcatccacaattttaaaagaaaaggggggattggggggtac
agtgcaggggaaagaatagtagacataatagcaacagacatacaaactaaagaattacaaaaacaaattacaaa
aattcaaaattttcgggtttattacagggacagcagagatccagtttggactagtcgtgaggctccggtgcccgtcagtg
ggcagagcgcacatcgcccacagtccccgagaagttggggggaggggtcggcaattgaaccggtgcctagagaa
ggtggcgcggggtaaactgggaaagtgatgtcgtgtactggctccgcctttttcccgagggtgggggagaaccgtata
taagtgcagtagtcgccgtgaacgttctttttcgcaacgggtttgccgccagaacacaggtaagtgccgtgtgtggttcc
cgcgggcctggcctctttacgggttatggcccttgcgtgccttgaattacttccacgcccctggctgcagtacgtgattcttg
atcccgagcttcgggttggaagtgggtgggagagttcgaggccttgcgcttaaggagccccttcgcctcgtgcttgagtt
gaggcctggcctgggcgctggggccgccgcgtgcgaatctggtggcaccttcgcgcctgtctcgctgctttcgataagt
ctctagccatttaaaatttttgatgacctgctgcgacgctttttttctggcaagatagtcttgtaaatgcgggccaagatctgc
acactggtatttcggtttttggggccgcgggcggcgacggggcccgtgcgtcccagcgcacatgttcggcgaggcgg
ggcctgcgagcgcggccaccgagaatcggacgggggtagtctcaagctggccggcctgctctggtgcctggcctcg
cgccgccgtgtatcgccccgccctgggcggcaaggctggcccggtcggcaccagttgcgtgagcggaaagatggc
cgcttcccggccctgctgcagggagctcaaaatggaggacgcggcgctcgggagagcgggcgggtgagtcaccc
acacaaaggaaaagggcctttccgtcctcagccgtcgcttcatgtgactccacggagtaccgggcgccgtccaggca
cctcgattagttctcgagcttttggagtacgtcgtctttaggttggggggaggggttttatgcgatggagtttccccacactg
agtgggtggagactgaagttaggccagcttggcacttgatgtaattctccttggaatttgccctttttgagtttggatcttggtt
cattctcaagcctcagacagtggttcaaagtttttttcttccatttcaggtgtcgtgaagcggccgcGCCACCATGA
AAGAAATCACTGTTGCTACATCACGAACGCTTTCTTATTACAAATTGGGAGCTTC
GCAGGGTAAAATGAAAGATCTCAGTCCAAGATGGTATTTCTACTACCTAGGAAC
TGGGCCAGAAGGCATCATATGGGTTGCAACTGAGGGAGCCTTGAATACACCAA
AAGATCACATTGGCACCGGGAGCAGAGGCGGCAGTCAAGCCTCTTCTCGTTCC
TCATCACGTAGTCGCAACAGTTCAATGTCTGGTAAAGGCCAACAACAACAAGGC

CAAACTGTCACTAAGAAATCTGCTGCTGAGCAATTTGCCCCCAGCGCTTCAGCG
TTCTTCGGAATGTCGCGCATTGGCATGGAAGTCAATAAGCATATTGACGCATAC
AAAACATTCCCACCAACAGAGCCTAAAAAGGACAAAAAGAAGAAGGTGGTCAAC
CAAAATGCACAAGCTTTAAACACGCTTGTTAAACAACTTAGCTCCAATTTTGGTA
CAATTATGCTTTGCTGTATGACCAGTTGCTGTAGTTGTCTCAAGGGCTGTTGTTC
TTGTACCGGTTATCCGTACGACGTGCCTGATTATGCCTGAtctagacccccccctaac
gttactggccgaagccgcttggaataaggccggtgtgcgtttgtctatatgttattttccaccatattgccgtcttttggcaat
gtgagggcccggaaacctggccctgtcttcttgacgagcattcctaggggtctttcccctctcgccaaaggaatgcaag
gtctgttgaatgtcgtgaaggaagcagttcctctggaagcttcttgaagacaaacaacgtctgtagcgaccctttgcagg
cagcggaaccccccacctggcgacaggtgcctctgcggccaaaagccacgtgtataagatacacctgcaaaggcg
gcacaaccccagtgccacgttgtgagttggatagttgtggaaagagtcaaatggctctcctcaagcgtattcaacaag
gggctgaaggatgcccagaaggtaccccattgtatgggatctgatctggggcctcggtgcacatgctttacatgtgttta
gtcgaggttaaaaaaacgtctaggccccccgaaccacggggacgtggttttcctttgaaaaacacgataataggatc
cATGACCGAGTACAAGCCCACGGTGCGCCTCGCCACCCGCGACGACGTCCCCA
GGGCCGTACGCACCCTCGCCGCCGCGTTCGCCGACTACCCCGCCACGCGCCA
CACCGTCGATCCGGACCGCCACATCGAGCGGGTCACCGAGCTGCAAGAACTCT
TCCTCACGCGCGTCGGGCTCGACATCGGCAAGGTGTGGGTCGCGGACGACGG
CGCCGCGGTGGCGGTCTGGACCACGCCGGAGAGCGTCGAAGCGGGGGCGGT
GTTCGCCGAGATCGGCCCGCGCATGGCCGAGTTGAGCGGTTCCCGGCTGGCC
GCGCAGCAACAGATGGAAGGCCTCCTGGCGCCGCACCGGCCCAAGGAGCCCG
CGTGGTTCCTGGCCACCGTCGGAGTCTCGCCCGACCACCAGGGCAAGGGTCT
GGGCAGCGCCGTCGTGCTCCCCGGAGTGGAGGCGGCCGAGCGCGCCGGGGT
GCCCGCCTTCCTGGAGACCTCCGCGCCCCGCAACCTCCCCTTCTACGAGCGGC
TCGGCTTCACCGTCACCGCCGACGTCGAGGTGCCCGAAGGACCGCGCACCTG
GTGCATGACCCGCAAGCCCGGTGCCTGAgctagcatcgatagatcctaatcaacctctggattac
aaaatttgtgaaagattgactggtattcttaactatgttgctccttttacgctatgtggatacgctgctttaatgcctttgtatcat
gctattgcttcccgtatggctttcattttctcctccttgtataaatcctggttgctgtctctttatgaggagttgtggcccgttgtca
ggcaacgtggcgtggtgtgcactgtgtttgctgacgcaacccccactggttggggcattgccaccacctgtcagctcctt
tccgggactttcgctttccccctccctattgccacggcggaactcatcgccgcctgccttgcccgctgctggacaggggc
tcggctgttgggcactgacaattccgtggtgttgtcggggaaatcatcgtcctttccttggctgctcgcctgtgttgccacct
ggattctgcgcgggacgtccttctgctacgtcccttcggccctcaatccagcggaccttccttcccgcggcctgctgccg
gctctgcggcctcttccgcgtcttcgccttcgccctcagacgagtcggatctccctttgggccgcctccccgcctgagatc
ctttaagaccaatgacttacaaggcagctgtagatcttagccactttttaaaagaaaaggggggactggaagggctaa
ttcactcccaacgaagacaagatctgctttttgcttgtactgggtctctctggttagaccagatctgagcctgggagctctct
ggctaactagggaacccactgcttaagcctcaataaagcttgccttgagtgcttcaagtagtgtgtgcccgtctgttgtgt
gactctggtaactagagatccctcagacccttttagtcagtgtggaaaatctctagcagtagtagttcatgtcatcttattatt
cagtatttataacttgcaaagaaatgaatatcagagagtgagaggcccgggttaattaaggaaagggctagatcattc
ttgaagacgaaagggcctcgtgatacgcctatttttataggttaatgtcatgataataatggtttcttagacgtcaggtggc
acttttcggggaaatgtgcgcggaacccctatttgtttatttttctaaatacattcaaatatgtatccgctcatgagacaataa
ccctgataaatgcttcaataatattgaaaaaggaagagtatgagtattcaacatttccgtgtcgcccttattcccttttttgcg
gcattttgccttcctgtttttgctcacccagaaacgctggtgaaagtaaaagatgctgaagatcagttgggtgcacgagtg
ggttacatcgaactggatctcaacagcggtaagatccttgagagttttcgccccgaagaacgttttccaatgatgagca
cttttaaagttctgctatgtggcgcggtattatcccgtgttgacgccgggcaagagcaactcggtcgccgcatacactatt
ctcagaatgacttggttgagtactcaccagtcacagaaaagcatcttacggatggcatgacagtaagagaattatgca

gtgctgccataaccatgagtgataacactgcggccaacttacttctgacaacgatcggaggaccgaaggagctaacc
gctttttgcacaacatgggggatcatgtaactcgccttgatcgttgggaaccggagctgaatgaagccataccaaacg
acgagcgtgacaccacgatgcctgtagcaatggcaacaacgttgcgcaaactattaactggcgaactacttactctag
cttcccggcaacaattaatagactggatggaggcggataaagttgcaggaccacttctgcgctcggcccttccggctg
gctggtttattgctgataaatctggagccggtgagcgtgggtctcgcggtatcattgcagcactggggccagatggtaa
gccctcccgtatcgtagttatctacacgacggggagtcaggcaactatggatgaacgaaatagacagatcgctgaga
taggtgcctcactgattaagcattggtaactgtcagaccaagtttactcatatatactttagattgatttaaaacttcattttta
atttaaaaggatctaggtgaagatcctttttgataatctcatgaccaaaatcccttaacgtgagttttcgttccactgagcgt
cagaccccgtagaaaagatcaaaggatcttcttgagatcctttttttctgcgcgtaatctgctgcttgcaaacaaaaaaa
ccaccgctaccagcggtggtttgtttgccggatcaagagctaccaactctttttccgaaggtaactggcttcagcagagc
gcagataccaaatactgttcttctagtgtagccgtagttaggccaccacttcaagaactctgtagcaccgcctacatacc
tcgctctgctaatcctgttaccagtggctgctgccagtggcgataagtcgtgtcttaccgggttggactcaagacgatagt
taccggataaggcgcagcggtcgggctgaacggggggttcgtgcacacagcccagcttggagcgaacgacctaca
ccgaactgagatacctacagcgtgagctatgagaaagcgccacgcttcccgaagggagaaaggcggacaggtat
ccggtaagcggcagggtcggaacaggagagcgcacgagggagcttccagggggaaacgcctggtatctttatagt
cctgtcgggtttcgccacctctgacttgagcgtcgatttttgtgatgctcgtcaggggggcggagcctatggaaaaacgc
cagcaacgcggcctttttacggttcctggccttttgctggccttttgctcacatgttctttcctgcgttatcccctgattctgtgga
taaccgtattaccgcctttgagtgagctgataccgctcgccgcagccgaacgaccgagcgcagcgagtcagtgagc
gaggaagcggaagagcgcccaatacgcaaaccgcctctccccgcgcgttggccgattcattaatgcagcaagctc
atggctgactaattttttttatttatgcagaggccgaggccgcctcggcctctgagctattccagaagtagtgaggaggctt
ttttggaggcctaggcttttgcaaaaagctccccgtggcacgacaggtttcccgactggaaagcgggcagtgagcgc
aacgcaattaatgtgagttagctcactcattaggcaccccaggctttacactttatgcttccggctcgtatgttgtgtggaat
tgtgagcggataacaatttcacacaggaaacagctatgacatgattacgaatttcacaaataaagcatttttttcactgc
attctagttgtggtttgtccaaactcatcaatgtatcttatcatgtctggatcaactggataactcaagctaaccaaaatcat
cccaaacttcccaccccataccctattaccactgccaattacctgtggtttcatttactctaaacctgtgattcctctgaatta
ttttcattttaaagaaattgtatttgttaaatatgtactacaaacttagtagt

TCR-01-1 (for Jurkat)
tggaagggctaattcactcccaaagaagacaagatatccttgatctgtggatctaccacacacaaggctacttccctga
ttagcagaactacacaccagggccaggggtcagatatccactgacctttggatggtgctacaagctagtaccagttga
gccagataaggtagaagaggccaataaaggagagaacaccagcttgttacaccctgtgagcctgcatgggatgga
tgacccggagagagaagtgttagagtggaggtttgacagccgcctagcatttcatcacgtggcccgagagctgcatc
cggagtacttcaagaactgctgatatcgagcttgctacaagggactttccgctggggactttccagggaggcgtggcct
gggcgggactggggagtggcgagccctcagatcctgcatataagcagctgctttttgcctgtactgggtctctctggtta
gaccagatctgagcctgggagctctctggctaactagggaacccactgcttaagcctcaataaagcttgccttgagtgc
ttcaagtagtgtgtgcccgtctgttgtgtgactctggtaactagagatccctcagacccttttagtcagtgtggaaaatctct
agcagtggcgcccgaacagggacttgaaagcgaaagggaaaccagaggagctctctcgacgcaggactcggctt
gctgaagcgcgcacggcaagaggcgaggggcggcgactggtgagtacgccaaaaattttgactagcggaggcta
gaaggagagagatgggtgcgagagcgtcagtattaagcgggggagaattagatcgcgatgggaaaaaattcggtt
aaggccagggggaaagaaaaaatataaattaaaacatatagtatgggcaagcagggagctagaacgattcgcag
ttaatcctggcctgttagaaacatcagaaggctgtagacaaatactgggacagctacaaccatcccttcagacaggat
cagaagaacttagatcattatataatacagtagcaaccctctattgtgtgcatcaaaggatagagataaaagacacca

aggaagctttagacaagatagaggaagagcaaaacaaaagtaagaccaccgcacagcaagcggccggccgct
gatcttcagacctggaggaggagatatgagggacaattggagaagtgaattatataaatataaagtagtaaaaattg
aaccattaggagtagcacccaccaaggcaaagagaagagtggtgcagagagaaaaaagagcagtgggaatag
gagctttgttccttgggttcttgggagcagcaggaagcactatgggcgcagcgtcaatgacgctgacggtacaggcca
gacaattattgtctggtatagtgcagcagcagaacaatttgctgagggctattgaggcgcaacagcatctgttgcaactc
acagtctggggcatcaagcagctccaggcaagaatcctggctgtggaaagatacctaaaggatcaacagctcctgg
ggatttggggttgctctggaaaactcatttgcaccactgctgtgccttggaatgctagttggagtaataaatctctggaac
agatttggaatcacacgacctggatggagtgggacagagaaattaacaattacacaagcttaatacactccttaattg
aagaatcgcaaaaccagcaagaaaagaatgaacaagaattattggaattagataaatgggcaagtttgtggaattg
gtttaacataacaaattggctgtggtatataaaattattcataatgatagtaggaggcttggtaggtttaagaatagtttttgc
tgtactttctatagtgaatagagttaggcagggatattcaccattatcgtttcagacccacctcccaaccccgaggggac
ccgacaggcccgaaggaatagaagaagaaggtggagagagagacagagacagatccattcgattagtgaacgg
atctcgacggtatcgccgaattcacaaatggcagtattcatccacaattttaaaagaaaaggggggattggggggtac
agtgcaggggaaagaatagtagacataatagcaacagacatacaaactaaagaattacaaaaacaaattacaaa
aattcaaaattttcgggtttattacagggacagcagagatccagtttggactagtcgtgaggctccggtgcccgtcagtg
ggcagagcgcacatcgcccacagtccccgagaagttggggggaggggtcggcaattgaaccggtgcctagagaa
ggtggcgcggggtaaactgggaaagtgatgtcgtgtactggctccgcctttttcccgagggtgggggagaaccgtata
taagtgcagtagtcgccgtgaacgttctttttcgcaacgggtttgccgccagaacacaggtaagtgccgtgtgtggttcc
cgcgggcctggcctctttacgggttatggcccttgcgtgccttgaattacttccacgcccctggctgcagtacgtgattcttg
atcccgagcttcgggttggaagtgggtgggagagttcgaggccttgcgcttaaggagccccttcgcctcgtgcttgagtt
gaggcctggcctgggcgctggggccgccgcgtgcgaatctggtggcaccttcgcgcctgtctcgctgctttcgataagt
ctctagccatttaaaatttttgatgacctgctgcgacgctttttttctggcaagatagtcttgtaaatgcgggccaagatctgc
acactggtatttcggtttttggggccgcgggcggcgacggggcccgtgcgtcccagcgcacatgttcggcgaggcgg
ggcctgcgagcgcggccaccgagaatcggacgggggtagtctcaagctggccggcctgctctggtgcctggcctcg
cgccgccgtgtatcgccccgccctgggcggcaaggctggcccggtcggcaccagttgcgtgagcggaaagatggc
cgcttcccggccctgctgcagggagctcaaaatggaggacgcggcgctcgggagagcgggcgggtgagtcaccc
acacaaaggaaaagggcctttccgtcctcagccgtcgcttcatgtgactccacggagtaccgggcgccgtccaggca
cctcgattagttctcgagcttttggagtacgtcgtctttaggttggggggaggggttttatgcgatggagtttccccacactg
agtgggtggagactgaagttaggccagcttggcacttgatgtaattctccttggaatttgccctttttgagtttggatcttggtt
cattctcaagcctcagacagtggttcaaagtttttttcttccatttcaggtgtcgtgaagcggccgcgccaccATGGGC
TGCAGGCTGCTCTGCTGTGCGGTTCTCTGTCTCCTGGGAGCAGTTCCCATAGA
CACTGAAGTTACCCAGACACCAAAACACCTGGTCATGGGAATGACAAATAAGAA
GTCTTTGAAATGTGAACAACATATGGGGCACAGGGCTATGTATTGGTACAAGCA
GAAAGCTAAGAAGCCACCGGAGCTCATGTTTGTCTACAGCTATGAGAAACTCTC
TATAAATGAAAGTGTGCCAAGTCGCTTCTCACCTGAATGCCCCAACAGCTCTCT
CTTAAACCTTCACCTACACGCCCTGCAGCCAGAAGACTCAGCCCTGTATCTCTG
CGCCAGCAGCTCGACAGCGGGGGGGTGGAATGAGCAGTTCTTCGGGCCAGGG
ACACGGCTCACCGTGCTAGAGGACCTGAACAAGGTGTTCCCACCCGAAGTGGC
CGTCTTCGAACCATCAGAAGCAGAGATCTCCCACACCCAAAAGGCCACACTGGT
GTGCCTGGCCACAGGCTTCTTCCCCGACCACGTGGAGCTGAGCTGGTGGGTGA
ATGGGAAGGAGGTGCACAGTGGGGTCtGCACAGACCCGCAGCCCCTCAAGGAG
CAGCCCGCCCTCAATGACTCCAGATACTGCCTGAGCAGCCGCCTGAGGGTCTC
GGCCACCTTCTGGCAGAACCCCCGCAACCACTTCCGCTGTCAAGTCCAGTTCTA
CGGGCTCTCGGAGAATGACGAGTGGACCCAGGATAGGGCCAAACCCGTCACC

CAGATCGTCAGCGCCGAGGCCTGGGGTAGAGCAGACTGTGGCTTTACCTCGGT
GTCCTACCAGCAAGGGGTCCTGTCTGCCACCATCCTCTATGAGATCCTGCTAGG
GAAGGCCACCCTGTATGCTGTGCTGGTCAGCGCCCTTGTGTTGATGGCCATGG
TCAAGAGAAAGGATTTCcggcggaaacggGGTAAGCCTATCCCTAACCCTCTCCTCG
GTCTCGATTCTACGagcggaagcggagctactaacttcagcctgctgaagcaggctggagacgtggagg
agaaccctggacctATGCTCCTGTTGCTCATACCAGTGCTGGGGATGATTTTTGCCCT
GAGAGATGCCAGAGCCCAGTCTGTGAGCCAGCATAACCACCACGTAATTCTCTC
TGAAGCAGCCTCACTGGAGTTGGGATGCAACTATTCCTATGGTGGAACTGTTAA
TCTCTTCTGGTATGTCCAGTACCCTGGTCAACACCTTCAGCTTCTCCTCAAGTAC
TTTTCAGGGGATCCACTGGTTAAAGGCATCAAGGGCTTTGAGGCTGAATTTATA
AAGAGTAAATTCTCCTTTAATCTGAGGAAACCCTCTGTGCAGTGGAGTGACACA
GCTGAGTACTTCTGTGCCGTGAATGAAGGAGATGACAAGATCATCTTTGGAAAA
GGGACACGACTTCATATTCTCCCCaATATCCAGAACCCTGACCCTGCTGTCTAT
CAACTCCGGGACTCTAAATCCAGTGACAAGTCTGTCTGCCTATTCACCGATTTT
GATTCTCAAACAAATGTGTCACAAAGTAAGGATTCTGATGTGTATATCACAGACA
AAtgTGTGCTAGACATGAGGTCTATGGACTTCAAGAGCAACAGTGCTGTGGCCT
GGAGCAACAAATCTGACTTTGCATGTGCAAACGCCTTCAACAACAGCATTATTC
CAGAAGACACCTTCTTCCCCAGCCCAGAAAGTTCCTGTGATGTCAAGCTGGTCG
AGAAAAGCTTTGAAACAGATACGAACCTAAACTTTCAAAACCTGTCAGTGATTGG
GTTCCGAATCCTCCTCCTGAAAGTGGCCGGGTTTAATCTGCTCATGACGCTGCG
GCTGTGGTCCAGCTGAgctagctcgagtctagaggatccctcccccccccctaacgttactggccgaagc
cgcttggaataaggccggtgtgcgtttgtctatatgttattttccaccatattgccgtcttttggcaatgtgagggcccggaa
acctggccctgtcttcttgacgagcattcctaggggtctttcccctctcgccaaaggaatgcaaggtctgttgaatgtcgtg
aaggaagcagttcctctggaagcttcttgaagacaaacaacgtctgtagcgaccctttgcaggcagcggaaccccc
acctggcgacaggtgcctctgcggccaaaagccacgtgtataagatacacctgcaaaggcggcacaaccccagtg
ccacgttgtgagttggatagttgtggaaagagtcaaatggctctcctcaagcgtattcaacaaggggctgaaggatgc
ccagaaggtaccccattgtatgggatctgatctggggcctcggtgcacatgctttacatgtgtttagtcgaggttaaaaaa
acgtctaggccccccgaaccacggggacgtggttttcctttgaaaaacacgataataccatgaccatggcctcctccg
aggacgtcatcaaggagttcatgcgcttcaaggtgcgcatggagggctccgtgaacggccacgagttcgagatcga
gggcgagggcgagggccgcccctacgagggcacccagaccgccaagctgaaggtgaccaagggcggccccct
gcccttcgcctgggacatcctgtcccctcagttccagtacggctccaaggcctacgtgaagcaccccgccgacatccc
cgactacttgaagctgtccttccccgagggcttcaagtgggagcgcgtgatgaacttcgaggacggcggcgtggtga
ccgtgacccaggactcctccctgcaggacggcgagttcatctacaaggtgaagctgcgcggcaccaacttcccctcc
gacggccccgtaatgcagaagaagaccatgggctgggaggcctccaccgagcggatgtaccccgaggacggcg
ccctgaagggcgagatcaagatgaggctgaagctgaaggacggcggccactacgacgccgaggtcaagaccac
ctacatggccaagaagcccgtgcagctgcccggcgcctacaagaccgacatcaagctggacatcacctcccacaa
cgaggactacaccatcgtggaacagtacgagcgcgccgagggccgccactccaccggcgcctaaatcgatagatc
ctaatcaacctctggattacaaaatttgtgaaagattgactggtattcttaactatgttgctccttttacgctatgtggatacgc
tgctttaatgcctttgtatcatgctattgcttcccgtatggctttcattttctcctccttgtataaatcctggttgctgtctctttatgag
gagttgtggcccgttgtcaggcaacgtggcgtggtgtgcactgtgtttgctgacgcaacccccactggttggggcattgc
caccacctgtcagctcctttccgggactttcgctttccccctccctattgccacggcggaactcatcgccgcctgccttgcc
cgctgctggacaggggctcggctgttgggcactgacaattccgtggtgttgtcggggaaatcatcgtcctttccttggctg
ctcgcctgtgttgccacctggattctgcgcgggacgtccttctgctacgtcccttcggccctcaatccagcggaccttcctt
cccgcggcctgctgccggctctgcggcctcttccgcgtcttcgccttcgccctcagacgagtcggatctccctttgggcc

gcctccccgcctgagatcctttaagaccaatgacttacaaggcagctgtagatcttagccactttttaaaagaaaaggg
gggactggaagggctaattcactcccaacgaagacaagatctgctttttgcttgtactgggtctctctggttagaccagat
ctgagcctgggagctctctggctaactagggaacccactgcttaagcctcaataaagcttgccttgagtgcttcaagtag
tgtgtgcccgtctgttgtgtgactctggtaactagagatccctcagacccttttagtcagtgtggaaaatctctagcagtagt
agttcatgtcatcttattattcagtatttataacttgcaaagaaatgaatatcagagagtgagaggcccgggttaattaag
gaaagggctagatcattcttgaagacgaaagggcctcgtgatacgcctatttttataggttaatgtcatgataataatggtt
tcttagacgtcaggtggcacttttcggggaaatgtgcgcggaacccctatttgtttatttttctaaatacattcaaatatgtatc
cgctcatgagacaataaccctgataaatgcttcaataatattgaaaaaggaagagtatgagtattcaacatttccgtgtc
gcccttattcccttttttgcggcattttgccttcctgtttttgctcacccagaaacgctggtgaaagtaaaagatgctgaagat
cagttgggtgcacgagtgggttacatcgaactggatctcaacagcggtaagatccttgagagttttcgccccgaagaa
cgttttccaatgatgagcacttttaaagttctgctatgtggcgcggtattatcccgtgttgacgccgggcaagagcaactc
ggtcgccgcatacactattctcagaatgacttggttgagtactcaccagtcacagaaaagcatcttacggatggcatga
cagtaagagaattatgcagtgctgccataaccatgagtgataacactgcggccaacttacttctgacaacgatcggag
gaccgaaggagctaaccgcttttttgcacaacatgggggatcatgtaactcgccttgatcgttgggaaccggagctga
atgaagccataccaaacgacgagcgtgacaccacgatgcctgtagcaatggcaacaacgttgcgcaaactattaac
tggcgaactacttactctagcttcccggcaacaattaatagactggatggaggcggataaagttgcaggaccacttctg
cgctcggcccttccggctggctggtttattgctgataaatctggagccggtgagcgtgggtctcgcggtatcattgcagca
ctggggccagatggtaagccctcccgtatcgtagttatctacacgacggggagtcaggcaactatggatgaacgaaa
tagacagatcgctgagataggtgcctcactgattaagcattggtaactgtcagaccaagtttactcatatatactttagatt
gatttaaaacttcatttttaatttaaaaggatctaggtgaagatcctttttgataatctcatgaccaaaatcccttaacgtgag
ttttcgttccactgagcgtcagaccccgtagaaaagatcaaaggatcttcttgagatcctttttttctgcgcgtaatctgctgc
ttgcaaacaaaaaaaccaccgctaccagcggtggtttgtttgccggatcaagagctaccaactctttttccgaaggtaa
ctggcttcagcagagcgcagataccaaatactgttcttctagtgtagccgtagttaggccaccacttcaagaactctgta
gcaccgcctacatacctcgctctgctaatcctgttaccagtggctgctgccagtggcgataagtcgtgtcttaccgggttg
gactcaagacgatagttaccggataaggcgcagcggtcgggctgaacggggggttcgtgcacacagcccagcttg
gagcgaacgacctacaccgaactgagatacctacagcgtgagctatgagaaagcgccacgcttcccgaagggag
aaaggcggacaggtatccggtaagcggcagggtcggaacaggagagcgcacgagggagcttccagggggaaa
cgcctggtatctttatagtcctgtcgggtttcgccacctctgacttgagcgtcgatttttgtgatgctcgtcaggggggcgga
gcctatggaaaaacgccagcaacgcggcctttttacggttcctggccttttgctggccttttgctcacatgttctttcctgcgt
tatcccctgattctgtggataaccgtattaccgcctttgagtgagctgataccgctcgccgcagccgaacgaccgagcg
cagcgagtcagtgagcgaggaagcggaagagcgcccaatacgcaaaccgcctctccccgcgcgttggccgattc
attaatgcagcaagctcatggctgactaatttttttatttatgcagaggccgaggccgcctcggcctctgagctattccag
aagtagtgaggaggcttttttggaggcctaggcttttgcaaaaagctccccgtggcacgacaggtttcccgactggaaa
gcgggcagtgagcgcaacgcaattaatgtgagttagctcactcattaggcaccccaggctttacactttatgcttccggc
tcgtatgttgtgtggaattgtgagcggataacaatttcacacaggaaacagctatgacatgattacgaatttcacaaata
aagcatttttttcactgcattctagttgtggtttgtccaaactcatcaatgtatcttatcatgtctggatcaactggataactcaa
gctaaccaaaatcatcccaaacttcccaccccataccctattaccactgccaattacctgtggtttcatttactctaaacct
gtgattcctctgaattattttcattttaaagaaattgtatttgttaaatatgtactacaaacttagtagt

K562-EGFP-KTFPPTEPK (for K562)

tggaagggctaattcactcccaaagaagacaagatatccttgatctgtggatctaccacacacaaggctacttccctga
ttagcagaactacacaccagggccaggggtcagatatccactgacctttggatggtgctacaagctagtaccagttga

gccagataaggtagaagaggccaataaaggagagaacaccagcttgttacaccctgtgagcctgcatgggatgga
tgacccggagagagaagtgttagagtggaggtttgacagccgcctagcatttcatcacgtggcccgagagctgcatc
cggagtacttcaagaactgctgatatcgagcttgctacaagggactttccgctggggactttccagggaggcgtggcct
gggcgggactggggagtggcgagccctcagatcctgcatataagcagctgctttttgcctgtactgggtctctctggtta
gaccagatctgagcctgggagctctctggctaactagggaacccactgcttaagcctcaataaagcttgccttgagtgc
ttcaagtagtgtgtgcccgtctgttgtgtgactctggtaactagagatccctcagacccttttagtcagtgtggaaaatctct
agcagtggcgcccgaacagggacttgaaagcgaaagggaaaccagaggagctctctcgacgcaggactcggctt
gctgaagcgcgcacggcaagaggcgaggggcggcgactggtgagtacgccaaaaattttgactagcggaggcta
gaaggagagagatgggtgcgagagcgtcagtattaagcgggggagaattagatcgcgatgggaaaaaattcggtt
aaggccagggggaaagaaaaaatataaattaaaacatatagtatgggcaagcagggagctagaacgattcgcag
ttaatcctggcctgttagaaacatcagaaggctgtagacaaatactgggacagctacaaccatcccttcagacaggat
cagaagaacttagatcattatataatacagtagcaaccctctattgtgtgcatcaaaggatagagataaaagacacca
aggaagctttagacaagatagaggaagagcaaaacaaaagtaagaccaccgcacagcaagcggccggccgct
gatcttcagacctggaggaggagatatgagggacaattggagaagtgaattatataaatataaagtagtaaaaattg
aaccattaggagtagcacccaccaaggcaaagagaagagtggtgcagagagaaaaaagagcagtgggaatag
gagctttgttccttgggttcttgggagcagcaggaagcactatgggcgcagcgtcaatgacgctgacggtacaggcca
gacaattattgtctggtatagtgcagcagcagaacaatttgctgagggctattgaggcgcaacagcatctgttgcaactc
acagtctggggcatcaagcagctccaggcaagaatcctggctgtggaaagatacctaaaggatcaacagctcctgg
ggatttggggttgctctggaaaactcatttgcaccactgctgtgccttggaatgctagttggagtaataaatctctggaac
agatttggaatcacacgacctggatggagtgggacagagaaattaacaattacacaagcttaatacactccttaattg
aagaatcgcaaaaccagcaagaaaagaatgaacaagaattattggaattagataaatgggcaagtttgtggaattg
gtttaacataacaaattggctgtggtatataaaattattcataatgatagtaggaggcttggtaggtttaagaatagtttttgc
tgtactttctatagtgaatagagttaggcagggatattcaccattatcgtttcagacccacctcccaaccccgaggggac
ccgacaggcccgaaggaatagaagaagaaggtggagagagagacagagacagatccattcgattagtgaacgg
atctcgacggtatcgccgaattcacaaatggcagtattcatccacaattttaaaagaaaaggggggattggggggtac
agtgcaggggaaagaatagtagacataatagcaacagacatacaaactaaagaattacaaaaacaaattacaaa
aattcaaaattttcgggtttattacagggacagcagagatccagtttggactagtcgtgaggctccggtgcccgtcagtg
ggcagagcgcacatcgcccacagtccccgagaagttggggggaggggtcggcaattgaaccggtgcctagagaa
ggtggcgcggggtaaactgggaaagtgatgtcgtgtactggctccgcctttttcccgagggtgggggagaaccgtata
taagtgcagtagtcgccgtgaacgttctttttcgcaacgggtttgccgccagaacacaggtaagtgccgtgtgtggttcc
cgcgggcctggcctctttacgggttatggcccttgcgtgccttgaattacttccacgcccctggctgcagtacgtgattcttg
atcccgagcttcgggttggaagtgggtgggagagttcgaggccttgcgcttaaggagccccttcgcctcgtgcttgagtt
gaggcctggcctgggcgctggggccgccgcgtgcgaatctggtggcaccttcgcgcctgtctcgctgctttcgataagt
ctctagccatttaaaatttttgatgacctgctgcgacgctttttttctggcaagatagtcttgtaaatgcgggccaagatctgc
acactggtatttcggtttttggggccgcgggcggcgacggggcccgtgcgtcccagcgcacatgttcggcgaggcgg
ggcctgcgagcgcggccaccgagaatcggacgggggtagtctcaagctggccggcctgctctggtgcctggcctcg
cgccgccgtgtatcgccccgccctgggcggcaaggctggcccggtcggcaccagttgcgtgagcggaaagatggc
cgcttcccggccctgctgcagggagctcaaaatggaggacgcggcgctcgggagagcgggcgggtgagtcaccc
acacaaaggaaaagggcctttccgtcctcagccgtcgcttcatgtgactccacggagtaccgggcgccgtccaggca
cctcgattagttctcgagcttttggagtacgtcgtctttaggttggggggaggggttttatgcgatggagtttccccacactg
agtgggtggagactgaagttaggccagcttggcacttgatgtaattctccttggaatttgccctttttgagtttggatcttggtt
cattctcaagcctcagacagtggttcaaagtttttttcttccatttcaggtgtcgtgaagcggccgcGCCACCatggtg
agcaagggcgaggagctgttcaccggggtggtgcccatcctggtcgagctggacggcgacgtaaacggccacaa
gttcagcgtgtccggcgagggcgagggcgatgccacctacggcaagctgaccctgaagttcatctgcaccaccggc

aagctgcccgtgccctggcccaccctcgtgaccaccctgacctacggcgtgcagtgcttcagccgctaccccgacca
catgaagcagcacgacttcttcaagtccgccatgcccgaaggctacgtccaggagcgcaccatcttcttcaaggacg
acggcaactacaagacccgcgccgaggtgaagttcgagggcgacaccctggtgaaccgcatcgagctgaagggc
atcgacttcaaggaggacggcaacatcctggggcacaagctggagtacaactacaacagccacaacgtctatatca
tggccgacaagcagaagaacggcatcaaggtgaacttcaagatccgccacaacatcgaggacggcagcgtgca
gctcgccgaccactaccagcagaacacccccatcggcgacggccccgtgctgctgcccgacaaccactacctgag
cacccagtccgccctgagcaaagaccccaacgagaagcgcgatcacatggtcctgctggagttcgtgaccgccgc
cgggatcactctcggcatggacgagctgtacaagagcggaagcggagctactaacttcagcctgctgaagcaggct
ggagacgtggaggagaaccctggacctAAAACATTCCCACCAACAGAGCCTAAAtaaatcgatag
atcctaatcaacctctggattacaaaatttgtgaaagattgactggtattcttaactatgttgctccttttacgctatgtggata
cgctgctttaatgcctttgtatcatgctattgcttcccgtatggctttcattttctcctccttgtataaatcctggttgctgtctcttat
gaggagttgtggcccgttgtcaggcaacgtggcgtggtgtgcactgtgtttgctgacgcaacccccactggttggggca
ttgccaccacctgtcagctcctttccgggactttcgctttccccctccctattgccacggcggaactcatcgccgcctgcctt
gcccgctgctggacaggggctcggctgttgggcactgacaattccgtggtgttgtcggggaaatcatcgtcctttccttgg
ctgctcgcctgtgttgccacctggattctgcgcgggacgtccttctgctacgtcccttcggccctcaatccagcggaccttc
cttcccgcggcctgctgccggctctgcggcctcttccgcgtcttcgccttcgccctcagacgagtcggatctccctttggg
ccgcctccccgcctgagatcctttaagaccaatgacttacaaggcagctgtagatcttagccactttttaaaagaaaag
gggggactggaagggctaattcactcccaacgaagacaagatctgctttttgcttgtactgggtctctctggttagacca
gatctgagcctgggagctctctggctaactagggaacccactgcttaagcctcaataaagcttgccttgagtgcttcaag
tagtgtgtgcccgtctgttgtgtgactctggtaactagagatccctcagacccttttagtcagtgtggaaaatctctagcagt
agtagttcatgtcatcttattattcagtatttataacttgcaaagaaatgaatatcagagagtgagaggcccgggttaatta
aggaaagggctagatcattcttgaagacgaaagggcctcgtgatacgcctatttttataggttaatgtcatgataataatg
gtttcttagacgtcaggtggcacttttcggggaaatgtgcgcggaacccctatttgtttatttttctaaatacattcaaatatgt
atccgctcatgagacaataaccctgataaatgcttcaataatattgaaaaaggaagagtatgagtattcaacatttccgt
gtcgcccttattcccttttttgcggcattttgccttcctgtttttgctcacccagaaacgctggtgaaagtaaaagatgctgaa
gatcagttgggtgcacgagtgggttacatcgaactggatctcaacagcggtaagatccttgagagttttcgccccgaag
aacgttttccaatgatgagcacttttaaagttctgctatgtggcgcggtattatcccgtgttgacgccgggcaagagcaac
tcggtcgccgcatacactattctcagaatgacttggttgagtactcaccagtcacagaaaagcatcttacggatggcat
gacagtaagagaattatgcagtgctgccataaccatgagtgataacactgcggccaacttacttctgacaacgatcgg
aggaccgaaggagctaaccgcttttttgcacaacatgggggatcatgtaactcgccttgatcgttgggaaccggagct
gaatgaagccataccaaacgacgagcgtgacaccacgatgcctgtagcaatggcaacaacgttgcgcaaactatt
aactggcgaactacttactctagcttcccggcaacaattaatagactggatggaggcggataaagttgcaggaccact
tctgcgctcggcccttccggctggctggtttattgctgataaatctggagccggtgagcgtgggtctcgcggtatcattgca
gcactggggccagatggtaagccctcccgtatcgtagttatctacacgacggggagtcaggcaactatggatgaacg
aaatagacagatcgctgagataggtgcctcactgattaagcattggtaactgtcagaccaagtttactcatatatacttta
gattgatttaaaacttcatttttaatttaaaaggatctaggtgaagatcctttttgataatctcatgaccaaaatcccttaacgt
gagttttcgttccactgagcgtcagaccccgtagaaaagatcaaaggatcttcttgagatcctttttttctgcgcgtaatctg
ctgcttgcaaacaaaaaaaccaccgctaccagcggtggtttgtttgccggatcaagagctaccaactctttttccgaag
gtaactggcttcagcagagcgcagataccaaatactgttcttctagtgtagccgtagttaggccaccacttcaagaactc
tgtagcaccgcctacatacctcgctctgctaatcctgttaccagtggctgctgccagtggcgataagtcgtgtcttaccgg
gttggactcaagacgatagttaccggataaggcgcagcggtcgggctgaacggggggttcgtgcacacagcccag
cttggagcgaacgacctacaccgaactgagatacctacagcgtgagctatgagaaagcgccacgcttcccgaagg
gagaaaggcggacaggtatccggtaagcggcagggtcggaacaggagagcgcacgagggagcttccaggggg
aaacgcctggtatctttatagtcctgtcgggtttcgccacctctgacttgagcgtcgatttttgtgatgctcgtcaggggggc

ggagcctatggaaaaacgccagcaacgcggcctttttacggttcctggccttttgctggccttttgctcacatgttctttcct
gcgttatcccctgattctgtggataaccgtattaccgcctttgagtgagctgataccgctcgccgcagccgaacgaccg
agcgcagcgagtcagtgagcgaggaagcggaagagcgcccaatacgcaaaccgcctctccccgcgcgttggcc
gattcattaatgcagcaagctcatggctgactaattttttttatttatgcagaggccgaggccgcctcggcctctgagctatt
ccagaagtagtgaggaggcttttttggaggcctaggcttttgcaaaaagctccccgtggcacgacaggtttcccgactg
gaaagcgggcagtgagcgcaacgcaattaatgtgagttagctcactcattaggcaccccaggctttacactttatgctt
ccggctcgtatgttgtgtggaattgtgagcggataacaatttcacacaggaaacagctatgacatgattacgaatttcac
aaataaagcatttttttcactgcattctagttgtggtttgtccaaactcatcaatgtatcttatcatgtctggatcaactggata
actcaagctaaccaaaatcatcccaaacttcccaccccataccctattaccactgccaattacctgtggtttcatttactct
aaacctgtgattcctctgaattattttcattttaaagaaattgtatttgttaaatatgtactacaaacttagtagt

Y-GAP-KTFPPTEPK (for yeast)
ATGCAGTTACTTCGCTGTTTTTCAATATTTTCTGTTATTGCTAGCGTTTTAGCAAA
AACATTCCCACCAACAGAGCCTAAAGGTGGTGGTGGTTCTGGTGGTGGTGGTT
CTGGTGGTGGTGGTTCTATTCAGAGGACTCCAAAAATTCAGGTCTACTCTAGAC
ATCCAGCTGAGAATGGTAAGTCTAACTTCTTGAACTGCTACGTATCTGGTTTCCA
TCCATCTGATATTGAAGTCGACTTGTTGAAAAACGGTGAGAGGATCGAAAAAGT
TGAGCACTCTGACTTGTCTTTTTCTAAGGACTGGTCCTTCTACTTGTTGTACTAC
ACCGAGTTCACTCCAACTGAAAAGGATGAATACGCTTGCAGGGTTAATCATGTC
ACTTTGTCCCAGCCAAAAATTGTCAAGTGGGACAGAGATATGGGTGGTGGTGGT
TCTGGTGGTGGTGGTTCTGGTGGTGGTGGTTCTGGTGGTGGTGGTTCTGGTTC
TCATTCTATGAGGTATTTCTACACCTCCGTGTCCCGGCCCGGCCGCGGGGAGC
CCCGCTTCATCGCCGTGGGCTACGTGGACGACACGCAGTTCGTGCGGTTCGAC
AGCGACGCCGCGAGCCAGAGGATGGAGCCGCGGGCGCCGTGGATAGAGCAG
GAGGGGCCGGAGTATTGGGACCAGGAGACACGGAATGTGAAGGCCCAGTCAC
AGACTGACCGAGTGGACCTGGGGACCCTGCGCGGCgctTACAACCAGAGCGAG
GACGGTTCTCACACCATCCAGATAATGTATGGCTGCGACGTGGGGCCGGACGG
GCGCTTCCTCCGCGGGTACCGGCAGGACGCCTACGACGGCAAGGATTACATC
GCCCTGAACGAGGACCTGCGCTCTTGGACCGCGGCGGACATGGCAGCTCAGA
TCACCAAGCGCAAGTGGGAGGCGGCCCATGCGGCGGAGCAGCAGAGAGCCTA
CCTGGAGGGCCGGTGCGTGGAGTGGCTCCGCAGATACCTGGAGAACGGGAAG
GAGACGCTGCAGCGCACGGACCCCCCCAAGACACATATGACCCACCACCCCAT
CTCTGACCATGAGGCCACCCTGAGGTGCTGGGCCCTGGGCTTCTACCCTGCGG
AGATCACACTGACCTGGCAGCGGGATGGGGAGGACCAGACCCAGGACACGGA
GCTCGTGGAGACCAGGCCTGCAGGGGATGGAACCTTCCAGAAGTGGGCGGCT
GTGGTGGTGCCTTCTGGAGAGGAGCAGAGATACACCTGCCATGTGCAGCATGA
GGGTCTGCCCAAGCCCCTCACCCTGAGATGGGAGCTGTCTTCCGGATCCTACC
CATACGACGTTCCAGACTACGCTAAGCTTCTGCAGGCTAGTGGTGGAGGAGGC
TCTGGTGGAGGCGGTAGCGGAGGCGGAGGGTCGCAGGAACTGACAACTATAT
GCGAGCAAATCCCCTCACCAACTTTAGAATCGACGCCGTACTCTTTGTCAACGA
CTACTATTTTGGCCAACGGGAAGGCAATGCAAGGAGTTTTTGAATATTACAAATC
AGTAACGTTTGTCAGTAATTGCGGTTCTCACCCCTCAACAACTAGCAAAGGCAG
CCCCATAAACACACAGTATGTTTTTTAA
