# Supplementary material for: Identification of virus epitopes and reactive T-cell receptors from memory T cells without peptide synthesis
Source: Commun Biol. 2024 Nov 4;7:1432. doi: 10.1038/s42003-024-07048-x (PMC11535475; doi:10.1038/s42003-024-07048-x)
Supplement: Supplementary file 2 — Description of Additional Supplementary Files [file 42003_2024_7048_MOESM2_ESM.pdf]

Description of Additional Supplementary Files

File name: Supplementary Data 1

Description: SARS-CoV-2 peptidome and presentation predictions for 139 HLA alleles.

File name: Supplementary Data 2

Description: SARS-CoV-2 sequences included in TMGs.

File name: Supplementary Data 3

Description: Donor information.

File name: Supplementary Data 4

Description: TCR sequences of engineered Jurkat T cells.
