# Supplementary material for: Identification of virus epitopes and reactive T-cell receptors from memory T cells without peptide synthesis
Source: Commun Biol. 2024 Nov 4;7:1432. doi: 10.1038/s42003-024-07048-x (PMC11535475; doi:10.1038/s42003-024-07048-x)
Supplement: Supplementary file 8 — Reporting Summary [file 42003_2024_7048_MOESM8_ESM.pdf]

nature portfolio

Corresponding author(s): Xun Lan, Xin Lin, Ai-long Huang,Ni Tang, Fei Gu

Last updated by author(s): Oct 3, 2024

Reporting Summary

Nature Portfolio wishes to improve the reproducibility of the work that we publish. This form provides structure for consistency and transparency in reporting. For further information on Nature Portfolio policies, see our Editorial Policies and the Editorial Policy Checklist.

Statistics

For all statistical analyses, confirm that the following items are present in the figure legend, table legend, main text, or Methods section.

n/a	Confirmed	
☐	☒	The exact sample size (*n*) for each experimental group/condition, given as a discrete number and unit of measurement
☐	☒	A statement on whether measurements were taken from distinct samples or whether the same sample was measured repeatedly
☐	☒	The statistical test(s) used AND whether they are one- or two-sided<br>*Only common tests should be described solely by name; describe more complex techniques in the Methods section.*
☐	☒	A description of all covariates tested
☐	☒	A description of any assumptions or corrections, such as tests of normality and adjustment for multiple comparisons
☐	☒	A full description of the statistical parameters including central tendency (e.g. means) or other basic estimates (e.g. regression coefficient) AND variation (e.g. standard deviation) or associated estimates of uncertainty (e.g. confidence intervals)
☐	☒	For null hypothesis testing, the test statistic (e.g. *F*, *t*, *r*) with confidence intervals, effect sizes, degrees of freedom and *P* value noted<br>*Give P values as exact values whenever suitable.*
☒	☐	For Bayesian analysis, information on the choice of priors and Markov chain Monte Carlo settings
☐	☒	For hierarchical and complex designs, identification of the appropriate level for tests and full reporting of outcomes
☐	☒	Estimates of effect sizes (e.g. Cohen's *d*, Pearson's *r*), indicating how they were calculated

Our web collection on statistics for biologists contains articles on many of the points above.

Software and code

Policy information about availability of computer code

Data collection	illumina HiSeq X Ten and NovaSeq 6000 System to generate the sequencing data.
Data analysis	Flow cytometry data was analyzed using FlowJo v10.<br>Transcriptome data was analyzed using BCL2fastq v2.20 (https://support.illumina.com/downloads/bcl2fastq-conversion-software-v2-20.html), Cell Ranger (https://support.10xgenomics.com/single-cell-gene-expression/software/release-notes/3-1, version 3.1), R (https://www.r-project.org/, version 4.0.1), R Studio (https://www.rstudio.com/), Python (https://www.python.org/,version 3.6.7), Scanpy(https://scanpy.readthedocs.io/en/stable/, version 1.8.2).

For manuscripts utilizing custom algorithms or software that are central to the research but not yet described in published literature, software must be made available to editors and reviewers. We strongly encourage code deposition in a community repository (e.g. GitHub). See the Nature Portfolio guidelines for submitting code & software for further information.

Data

Policy information about availability of data

All manuscripts must include a data availability statement. This statement should provide the following information, where applicable:

- Accession codes, unique identifiers, or web links for publicly available datasets
- A description of any restrictions on data availability
- For clinical datasets or third party data, please ensure that the statement adheres to our policy

The processed expression matrix, TCR information, and cell annotations in this paper are available in OMIX database with accession ID: OMIX001069, https://ngdc.cncb.ac.cn/omix/release/OMIX001069; The raw data of single-cell sequencing have been deposited into GSA-human database with accession ID: HRA002230, https://ngdc.cncb.ac.cn/gsa-human/browse/HRA002230

Research involving human participants, their data, or biological material

Policy information about studies with human participants or human data. See also policy information about sex, gender (identity/presentation), and sexual orientation and race, ethnicity and racism.

Reporting on sex and gender	The gender data of all donors enrolled in the article was collected; for details, see Data File S3. In accordance with ethical requirements, we recruited a total of 35 individuals from Tsinghua University (Project No: 20210030) and Chongqing Medical University (Project No: 2021066). All participants signed informed consent forms, including consent for the sharing of individual-level data. We specifically targeted 31 donors carrying the HLA-A*11:01 allele, the most prevalent HLA-A allele among Chinese individuals. Among them, 13 individuals unexposed to COVID-19 included 8 females and 5 males. Among 18 recovered donors at 7-8 months post-infection, there were 7 females and 11 males. Our findings are not applicable to only one gender. The gender of participants was determined based on self-report.<br>We didn't report gender-based analyses because our study aimed to screen TCR sequences using the recruited population and to successfully demonstrate the feasibility of our method. Although our cohort includes both males and females, the differences in TCR sequences between genders are not the focus of our research.
Reporting on race, ethnicity, or other socially relevant groupings	This study does not involve race, ethnicity, or other socially relevant groupings.
Population characteristics	13 donors unexposed to COVID-19 and 18 recovered donors 7-8 months after the infection of COVID-19 were enrolled in this study. Detailed information can be found in the Date File S3.
Recruitment	All donors were recruited and managed without any specific bias by Yongchuan Hospital of Chongqing Medical University and Tsinghua University.
Ethics oversight	This project was approved by the ethics committee of Tsinghua University (Project No: 20210030) and Chongqing Medical University (Project No: 2021066).

Note that full information on the approval of the study protocol must also be provided in the manuscript.

Field-specific reporting

Please select the one below that is the best fit for your research. If you are not sure, read the appropriate sections before making your selection.

☒ Life sciences ☐ Behavioural & social sciences ☐ Ecological, evolutionary & environmental sciences

For a reference copy of the document with all sections, see nature.com/documents/nr-reporting-summary-flat.pdf

Life sciences study design

All studies must disclose on these points even when the disclosure is negative.

Sample size	Sample size for the 10XGenomics scRNA-seq and TCR sequencing was determined by the availability of donors' samples. No statistical tests were performed for the sample size calculation but it was sufficient for this proof-of-concept study. The exact number of samples used per figure is informed in each figure.
Data exclusions	For single-cell sequencing data, cells with no TCR sequence, less than 400 UMI counts, less than 200 genes, or greater than 10% of mitochondrial gene counts were removed.
Replication	All attempts at replication were successful.
Randomization	The recovered donors and unexposed donors were recruited randomly in this study.
Blinding	Investigators were blinded to donor identities.

Reporting for specific materials, systems and methods

We require information from authors about some types of materials, experimental systems and methods used in many studies. Here, indicate whether each material, system or method listed is relevant to your study. If you are not sure if a list item applies to your research, read the appropriate section before selecting a response.

Materials & experimental systems

n/a	Involved in the study
☐	☒ Antibodies
☐	☒ Eukaryotic cell lines
☒	☐ Palaeontology and archaeology
☒	☐ Animals and other organisms
☒	☐ Clinical data
☒	☐ Dual use research of concern
☒	☐ Plants

Methods

n/a	Involved in the study
☒	☐ ChIP-seq
☐	☒ Flow cytometry
☒	☐ MRI-based neuroimaging

Antibodies

Antibodies used	All antibodies were commercially purchased and included: anti-human CD3-PB(Biolegend,cat#317313),anti-human CD8-APC(Biolegend,cat#301014),anti-human CD4-FITC(Biolegend), anit-4-1BB-PE(Biolegend,cat#309803), anti-human CD69-APC(Biolegend,cat#310910),Human IFN-γ pre-coated ELSPOT kit (Dakewe,cat#2110005)
Validation	All the antibodies used in this study were commercial antibodies, with validation procedures described on the following sites of the manufacturers:<br>anti-human CD3-PB(Biolegend,cat#317313)<br>https://www.biolegend.com/en-us/products/pacific-blue-anti-human-cd3-antibody-3648<br>anti-human CD8a-APC(Biolegend,cat#301014)<br>https://www.biolegend.com/en-us/products/apc-anti-human-cd8a-antibody-831<br>anti-human CD4-FITC(Biolegend,cat#317407)<br>https://www.biolegend.com/en-us/products/fitc-anti-human-cd4-antibody-3653<br>anit-4-1BB-PE(Biolegend,cat#309803)<br>https://www.biolegend.com/en-us/products/pe-anti-human-cd137-4-1bb-antibody-1510<br>anti-human CD69-APC(Biolegend,cat#310910)<br>https://www.biolegend.com/en-us/products/apc-anti-human-cd69-antibody-1674<br>Human IFN-γ pre-coated ELSPOT kit (Dakewe,cat#2110005)<br>http://bio-city.net/dayou/2110005.pdf

Eukaryotic cell lines

Policy information about cell lines and Sex and Gender in Research

Cell line source(s)	Jurkat clone 5 (JC5) cells were derived from Jurkat E6.1 cells (ATCC TIB-152) by knocking out TCRα and TCRβ chains with a CRISPR/Cas9 system (gRNA sequences: TRBC_GGGCTCAAACACAGCGACCTC, TRAC_GTCTCTCAGCTGGTACACGGC). K562 cell line (ATCC CCL-243) was used to expressing HLA-A*1101 and the epitopes. These cell lines were constructed by Dr. Xin Lin's lab from Tsinghua University, Beijing, China. The yeast strain EBY100α is a gift from Eric Klavins's lab.
Authentication	These original cell lines were authenticated by ATCC.
Mycoplasma contamination	Mycoplasma contamination was negative based on the PCR results using the supernatant of the culture medium.
Commonly misidentified lines<br>(See ICLAC register)	No commonly misidentified cell lines were used.

Plants

Seed stocks: NA

Novel plant genotypes: NA

Authentication: NA

Flow Cytometry

Plots

Confirm that:

☒ The axis labels state the marker and fluorochrome used (e.g. CD4-FITC).

☒ The axis scales are clearly visible. Include numbers along axes only for bottom left plot of group (a 'group' is an analysis of identical markers).

☒ All plots are contour plots with outliers or pseudocolor plots.

☒ A numerical value for number of cells or percentage (with statistics) is provided.

Methodology

Sample preparation: Information provided in Methods section

Instrument: LSRFortessa flow cytometer

Software: FlowJ software V10

Cell population abundance: Up to 10^4 single viable cells were sorted from each PBMC

Gating strategy: Information available on Table S1, Fig. S2,S3,S6 and Methods sections.

☒ Tick this box to confirm that a figure exemplifying the gating strategy is provided in the Supplementary Information.
